# Supplementary material for: Transcriptomes of Injured Lamprey Axon Tips: Single-Cell RNA-Seq Suggests Differential Involvement of MAPK Signaling Pathways in Axon Retraction and Regeneration after Spinal Cord Injury
Source: Cells. 2022 Jul 27;11(15):2320. doi: 10.3390/cells11152320 (PMC9367414; doi:10.3390/cells11152320)
Supplement: Supplementary file 1 [file cells-11-02320-s001.zip › cells-1803217-supplementary/cells-1803217supplementary/cells-1803217-figures and tables/Legends for supplemental data.pdf]

Figure S1. Determination of growing status of lamprey axon tips. Top and middle fluorescent images: determination of growing status by alignment. Bottom image: after micro-aspiration. Insets in bottom image: glass tips with fluorescent aspirate. Number on top left: animal ID with body length. **A,** Ten growing tips in 9 spinal cords; **B,** Nine static tips in 9 spinal cords; **C,** Five retracting tips in 5 spinal cords; **D,** Removed axon tips in 9 spinal cords due to ambiguous growing status, or repeated impalement.

Figure S2. Hierarchical cluster analysis of gene expression in growing, static, and retracting axon tips (full). **A,** 10 growing tips (G1-G10) vs. 5 retracting tips (R1-R5); **B,** 10 growing tips (G1-G10) *vs.* 9 static tips (S1-S9); **C,** 5 retracting tips (R1-R5) *vs.* 9 static tips (S1-S9).

Figure S3. GO enrichment analysis of DEGs in G *vs.* S tips. Genes that were differentially expressed between growing tips and static tips (151 genes), were analyzed by DAVID enrichment analysis. **A,** GO terms for genes expressed more highly in **G** tips than **S** tips (fold change FC > 1.5, FDR < 0.1). The horizontal axis represents the -log10(FDR) for the significant GO terms. The x-intercept of the vertical red line: FDR = 0.05. Pink term, pathway; Black terms, other cell function. **B,** modified volcano plot for genes expressed more highly in **G** tips than **S** tips ($p < 0.05$, FC > 2). Red dots, protein synthesis; Blue dots, modifications of DNA or chromosomes; Pink dot, MAPK pathway; Black dots, mitosis. It suggests **G** tips are more involved in protein synthesis than **S** tips are.

Figure S4. Principal Component Analysis of expressed transcripts. PCA was performed (cutoff > 5) to identify the sources of variance in our gene expression data from 24 axon tips with different growth statuses (**G**, **S** and **R**). Axon tips S-2 (blue dot at the top-right) and G-4 (red dot at the bottom) were identified as the main sources of variance. The remaining 22 tips (the group of dots in top-left region) showed relatively less variance, and clustered on the top-left area.

Figure S5. Genes coding for proteins involved in the regulation of histone function are identified in growing, static, and retracting axon tips. Genes obtained by RNA-seq from axoplasms of growing (**G**; 5,097), static (**S**; 4,105) and retracting tips (**R**; 2,318) are mapped in a Venn diagram and were analyzed by enrichment analysis (DAVID, v. 6.8). Genes involved in regulation of histone function are listed inside the gray frames. Red symbols are **histone acetyltransferase** (**hat**), and **histone deacetylases** (**hdac**). Pink symbols are the genes selected for validation in real-time q-PCR.

Figure S6. Western blot of proteins prepared from lamprey brain (Br) or spinal cord (SC), probed with antibodies against map3k2 (left), and csnk1e (right).
Left, colored ladders and number: protein size (Kda), imaged after protein was transferred; Right, fluorescence images showing labeled bands by antibodies against map3k2 and csnk1e.

Figure S7. Network analysis of gene expression related to csnk1e. Blue to yellow gradation and green color represent gene expression levels, from low to high. **Diamond, circle,** or **triangle nodes** represent genes that are predominant in growing, static, or retracting tips, respectively. The gray rectangular node spata32 is important (central), but it was not present in the sequencing lists. Enrichment analysis indicated that they are related to **circadian rhythms** (FDR = 7.14E-18)**.**

Table S1A. Ten growing tips, 9 static tips and 5 retracting tips were selected for DEG analysis. **Table S1B. Nine axon tips were excluded from the DEG analysis**.

Legends for Table S2-S3 are not available

Table S4. Pathways identified by 3 pathway databases, BBI, BIOCARTA, and KEGG during DAVID GO enrichment analysis. Genes from **Figure 3D** list A (G > S&R, 38 genes, **Table A**); list B (S > G&S, 20 genes, **Table B**); and list C (R > G&S, 18 genes, **Table C**) were analyzed on the DAVID GO enrichment analysis platform. Pathway(s) identified by BBI, BIOCARTA, or KEGG are listed for each gene. BBID identified only one pathway from two genes (mapsk2, mapk8); BIOCARTA identified 4 pathways from 4 genes (csnk1e, map3k2, mapk8, and prkcq, highlighted in pink); and KEGG generated more than 40 pathways from 26 genes.

Table S5. Related genes under important GO terms from DEGs of G *vs.* R tips. Genes in **group A** (1,488) were analyzed by DAVID. Sixty-five significant GO terms were generated based on FDR values (FDR< 0.05). Related genes are listed under important GO terms, including genes related to **cytoskeleton** (FDR = 7.69E-05), **mitochondria** (1.77E-06), **mRNA splicing** (0.03), **protein biosynthesis** (0.01), **ribosome biogenesis in eukaryotes** (0.03), **rRNA processing** (5.01E-04), **mRNA processing** (0.006), **tRNA processing** (0.005), **poly(A) RNA binding** (1.55E-05).

Table S6. Correlation analysis of counting data among G, S, and R groups. A, coefficients of determination (R^2) were calculated from paired transcriptomes: *e.g.*, G1,2 means the R^2 between columns G1 and G2 in **Table S2**, *etc.*. **B,** Comparison of coefficients of determination within G, S, and R groups, *e.g.*, G1,2 in **A** is 0.107, this value is assigned to the cell between row G1 and column G2, or column G1 and row G1. The sum of R^2 values is calculated in the right-hand column. The 5 most highly correlated transcriptomes were determined by the 5 highest R^2 values (highlighted in yellow).

Table S7. Raw data used in protein-protein interaction networks. **Top panel**: Data for plotting the map3k2 network. **Average (G)** column were obtained from Table S2, by calculating the means of G1 to G10 for the particular gene. The same procedure was

followed for the S columns (S1-S9), and the R columns (R1-R5). The shapes (diamond, circle, or triangle) in **Figure 11** are determined by the values of each row. A diamond indicates that the **Average (G)** value is highest, a circle if the **Average (S)** is highest, and a triangle if the **Average (R)** is highest. **Bottom panel**: The same calculations are designed for the csnk1e network (**Figure S7**).
