# Supplementary material for: Transcriptomes of Injured Lamprey Axon Tips: Single-Cell RNA-Seq Suggests Differential Involvement of MAPK Signaling Pathways in Axon Retraction and Regeneration after Spinal Cord Injury
Source: Cells. 2022 Jul 27;11(15):2320. doi: 10.3390/cells11152320 (PMC9367414; doi:10.3390/cells11152320)
Supplement: Supplementary file 1 [file cells-11-02320-s001.zip › cells-1803217-supplementary/cells-1803217supplementary/cells-1803217-figures and tables/Table S1AB (spinal cord).pdf]

Table S1A. Ten growing tips, 9 static tips and 5 retracting tips were selected for the DEG analysis.

Animal ID	Status	Tube	Sample Name
092520-8	Growing	29	G1
100220-1	Growing	35	G2
062620-4	Growing	3	G3
062620-6	Growing	4	G4
082720-10	Growing	12	G5
090720-8	Growing	14	G6
070320-4	Growing	8	G7
100220-1	Growing	34	G8
100220-2	Growing	38	G9
091120-1	Growing	15	G10
091820-3	Static	19	S 1
092520-7	Static	28	S 2
100220-1	Static	33	S 3
062620-2	Static	1	S 4
091820-8	Static	22	S 5
092520-10	static	30	S 6
070320-7	Static	9	S 7
082720-3	Static	10	S 8
091820-7	Static	21	S 9
062620-3	Retracting	2	R1
091820-10	Retracting	24	R2
070320-1	Retracting	7	R3
091820-9	Retracting	23	R4
091120-10	Retracting	18	R5

Table S1B. Nine axon tips were excluded for the DEG analysis. Tips were excluded from analysis either because on inspection of photomicrographs, they had grown or retracted > 3 μm but < 10 μm and thus their growth status was ambiguous, or their leading edge could not be identified with certainty, or because they had to be impaled twice in order to obtain sufficient axoplasm for analysis.

Deleted samples				
**Animal ID**	**Status**	**Tube**	**Sample Name**	**Notes**
092520-1	Growing?	25	G11	status ambiguous
090720-1	Growing	13	G12	impaled twice, possible contamination
092520-4	Growing?	27	G13	status ambiguous
092520-2	Growing?	26	G14	status ambiguous
091820-6	Growing	20	G15	impaled twice, possible contamination
082720-4	Growing?	11	G16	impaled twice, possible contamination
100220-1	Static?	37	S10	front edge not well defined
091120-4	Static	16	S11	impaled twice, possible contamination
091120-8	Retracting?	17	R6	status ambiguous
