# Supplementary material for: Transcriptomes of Injured Lamprey Axon Tips: Single-Cell RNA-Seq Suggests Differential Involvement of MAPK Signaling Pathways in Axon Retraction and Regeneration after Spinal Cord Injury
Source: Cells. 2022 Jul 27;11(15):2320. doi: 10.3390/cells11152320 (PMC9367414; doi:10.3390/cells11152320)
Supplement: Supplementary file 1 [file cells-11-02320-s001.zip › cells-1803217-supplementary/cells-1803217supplementary/cells-1803217-figures and tables/Table S4.pdf]

Table-A (G > S&R)

Entrez Gene-ID	Gene Name	BBID	BIOCARTA	KEGG_PATHWAY
607	BCL9 transcription coactivator(BCL9)			
55571	CCR4-NOT transcription complex subunit 11(CNOT11)			
51496	CTD small phosphatase like 2(CTDSPL2)			
3980	DNA ligase 3(LIG3)			hsa03410:Base excision repair,
57697	FA complementation group M(FANCM)			hsa03460:Fanconi anemia pathway,
60681	FKBP prolyl isomerase 10(FKBP10)			
60558	GTP binding elongation factor GUF1(GUF1)			
55127	HEAT repeat containing 1(HEATR1)			hsa03008:Ribosome biogenesis in eukaryotes,
284058	KAT8 regulatory NSL complex subunit 1(KANSL1)			
374354	NHL repeat containing 2(NHLRC2)			
23386	NudC domain containing 3(NUDCD3)			
5395	PMS1 homolog 2, mismatch repair system component(PMS2)			hsa03430:Mismatch repair,hsa03460:Fanconi anemia pathway,
29101	SSU72 homolog, RNA polymerase II CTD phosphatase(SSU72)			hsa03015:mRNA surveillance pathway,
9013	TATA-box binding protein associated factor, RNA polymerase I subunit C(TAF1C)			
64743	WD repeat domain 13(WDR13)			
84219	WD repeat domain 24(WDR24)			hsa04150:mTOR signaling pathway,
54432	Yip1 domain family member 1(YIPF1)			
9520	aminopeptidase puromycin sensitive(NPEPPS)			
1454	casein kinase 1 epsilon(CSNK1E)		h_circadianPathway:Circadian Rhythms,	hsa04710:Circadian rhythm, hsa04068:FoxO signaling pathway,hsa04310:Wnt signaling pathway,hsa04340:Hedgehog signaling, etc.
80125	coiled-coil domain containing 33(CCDC33)			
1036	cysteine dioxygenase type 1(CDO1)			hsa00270:Cysteine and methionine metabolism,hsa00430:Taurine and hypotaurine metabolism,hsa01100:Metabolic pathways, etc.
23332	cytoplasmic linker associated protein 1(CLASP1)			
1801	diphthamide biosynthesis 1(DPH1)			
55862	ethylmalonyl-CoA decarboxylase 1(ECHDC1)			hsa00640:Propanoate metabolism,hsa01100:Metabolic pathways,
84668	family with sequence similarity 126 member A(FAM126A)			
2968	general transcription factor IIH subunit 4(GTF2H4)			hsa03022:Basal transcription factors,hsa03420:Nucleotide excision repair,hsa05203:Viral carcinogenesis,
55726	integrator complex subunit 13(INTS13)			
64135	interferon induced with helicase C domain 1(IFIH1)			hsa04622:RIG-I-like receptor signaling pathway, hsa05161:Hepatitis B, hsa05162:Measles,hsa05164:Influenza A, etc.
144501	keratin 80(KRT80)			
10746	mitogen-activated protein kinase kinase kinase 2(MAP3K2)	100.MAPK_signaling_cascades,	h_mapkPathway:MAPKinase Signaling Pathway,	hsa04010:MAPK signaling pathway,hsa04540:Gap junction,hsa04912:GnRH signaling pathway,
7468	nuclear receptor binding SET domain protein 2(NSD2)			hsa00310:Lysine degradation,hsa01100:Metabolic pathways,hsa05202:Transcriptional misregulation in cancer,
25926	nucleolar protein 11(NOL11)			
9487	phosphatidylinositol glycan anchor biosynthesis class L(PIGL)			hsa00563:Glycosylphosphatidylinositol (GPI)-anchor biosynthesis,hsa01100:Metabolic pathways,
130535	potassium channel tetramerization domain containing 18(KCTD18)			
80162	protein-glucosylgalactosylhydroxylysine glucosidase(PGGHG)			
6608	smoothened, frizzled class receptor(SMO)		h_shhPathway:Sonic Hedgehog (Shh) Pathway,	hsa04340:Hedgehog signaling pathway, hsa04360:Axon guidance, hsa05200:Pathways in cancer,
85451	unk zinc finger(UNK)			
7707	zinc finger protein 148(ZNF148)			

Table-B (S > G&R)

Entrez Gene-ID	Gene Name	BBID	BIOCARTA	KEGG_PATHWAY
491	ATPase plasma membrane Ca2+ transporting 2(ATP2B2)			hsa04020:Calcium signaling pathway, hsa04022:cGMP-PKG signaling pathway, hsa04024:cAMP signaling pathway, etc.
27163	N-acylethanolamine acid amidase(NAAA)			
54726	OTU deubiquitinase 4(OTUD4)			
64902	alanine--glyoxylate aminotransferase 2(AGXT2)			hsa00250:Alanine, aspartate and glutamate metabolism, hsa01100:Metabolic pathways, etc.
8839	cellular communication network factor 5(CCN5)			
1277	collagen type I alpha 1 chain(COL1A1)			hsa04151:PI3K-Akt signaling pathway, hsa04510:Focal adhesion, hsa04512:ECM-receptor interaction, etc.
11068	cytochrome b561 family member D2(CYB561D2)			
89978	diphthamine biosynthesis 6(DPH6)			
9669	eukaryotic translation initiation factor 5B(EIF5B)			
10634	growth arrest specific 2 like 1(GAS2L1)			
5599	mitogen-activated protein kinase 8(MAPK8)	100.MAPK_signaling_cascades,101.[illegible]	h_dspPathway:Regulation of MAP Kinase Pathways Through	hsa04010:MAPK signaling pathway, hsa04012:ErbB signaling pathway, hsa04014:Ras signaling pathway, etc.
91775	neurexophilin and PC-esterase domain family member 3(NXPE3)			
27156	radial spoke head 14 homolog(RSPH14)			
54778	ring finger protein 111(RNF111)			
192683	secretory carrier membrane protein 5(SCAMP5)			
8991	selenium binding protein 1(SELENBP1)			hsa00920:Sulfur metabolism,hsa01100:Metabolic pathways,
57419	solute carrier family 24 member 3(SLC24A3)			
51000	solute carrier family 35 member B3(SLC35B3)			
6975	tectorin beta(TECTB)			
54957	thioredoxin like 4B(TXNL4B)			

Table-C (R > G&S)

Entrez Gene-ID	Gene Name	BBID	BIOCARTA	KEGG_PATHWAY
80067	DDB1 and CUL4 associated factor 17(DCAF17)			
4043	LDL receptor related protein associated protein 1(LRPAP1)			hsa04979:Cholesterol metabolism,
254956	MORN repeat containing 5(MORN5)			
100532724	NPHP3-ACAD11 readthrough (NMD candidate)(NPHP3-ACAD11)			
60561	RAD50 interactor 1(RINT1)			
84193	SET domain containing 3, actin histidine methyltransferase(SETD3)			
10629	TATA-box binding protein associated factor 6 like(TAF6L)			hsa03022:Basal transcription factors,
80315	cytoplasmic polyadenylation element binding protein 4(CPEB4)			hsa04114:Oocyte meiosis,hsa04914:Progesterone-mediated oocyte maturation,
1770	dynein axonemal heavy chain 9(DNAH9)			hsa05014:Amyotrophic lateral sclerosis,hsa05016:Huntington disease,hsa05022:Pathways of neurodegeneration - multiple diseases,
10691	glucocorticoid modulatory element binding protein 1(GMEB1)			
7184	heat shock protein 90 beta family member 1(HSP90B1)			hsa04141:Protein processing in endoplasmic reticulum, hsa04151:PI3K-Akt signaling pathway, etc.
84263	hydroxysteroid dehydrogenase like 2(HSDL2)			
9767	jade family PHD finger 3(JADE3)			
64779	methenyltetrahydrofolate synthetase domain containing(MTHFSD)			
79605	piggyBac transposable element derived 5(PGBD5)			
5588	protein kinase C theta(PRKCQ)		h_keratinocytePathway:Keratinocyte Differentiation,	hsa04064:NF-kappa B signaling pathway, hsa04660:T cell receptor signaling pathway, etc.
85415	rhophilin Rho GTPase binding protein 2(RHPN2)			
152579	sec1 family domain containing 2(SCFD2)			
