# Supplementary material for: Transcriptomes of Injured Lamprey Axon Tips: Single-Cell RNA-Seq Suggests Differential Involvement of MAPK Signaling Pathways in Axon Retraction and Regeneration after Spinal Cord Injury
Source: Cells. 2022 Jul 27;11(15):2320. doi: 10.3390/cells11152320 (PMC9367414; doi:10.3390/cells11152320)
Supplement: Supplementary file 1 [file cells-11-02320-s001.zip › cells-1803217-supplementary/cells-1803217supplementary/cells-1803217-figures and tables/Table S5.pdf]

Cytoskeleton

ENTREZ_GENE_ID	GENE NAME
54828	BCAS3, microtubule associated cell migration factor(BCAS3)
598	BCL2 like 1(BCL2L1)
26005	C2 calcium dependent domain containing 3(C2CD3)
23586	DExD/H-box helicase 58(DDX58)
23194	F-box and leucine rich repeat protein 7(FBXL7)
55691	FERM domain containing 4A(FRMD4A)
2242	FES proto-oncogene, tyrosine kinase(FES)
2314	FLII, actin remodeling protein(FLII)
85302	Fas binding factor 1(FBF1)
54801	HAUS augmin like complex subunit 6(HAUS6)
10198	M-phase phosphoprotein 9(MPHOSPH9)
4751	NIMA related kinase 2(NEK2)
134492	NudC domain containing 2(NUDCD2)
4218	RAB8A, member RAS oncogene family(RAB8A)
5888	RAD51 recombinase(RAD51)
84934	RBPJ interacting and tubulin associated 1(RITA1)
6093	Rho associated coiled-coil containing protein kinase 1(ROCK1)
8607	RuvB like AAA ATPase 1(RUVBL1)
8717	TNFRSF1A associated via death domain(TRADD)
11078	TRIO and F-actin binding protein(TRIOBP)
57728	WD repeat domain 19(WDR19)
10152	abl interactor 2(ABI2)
273	amphiphysin(AMPH)
54518	amyloid beta precursor protein binding family B member 1 interacting protein(APBB1IP)
1499	catenin beta 1(CTNNB1)
201161	centromere protein V(CENPV)
201134	centrosomal protein 112(CEP112)
145508	centrosomal protein 128(CEP128)
80254	centrosomal protein 63(CEP63)
10575	chaperonin containing TCP1 subunit 4(CCT4)
115948	coiled-coil domain containing 151(CCDC151)
23332	cytoplasmic linker associated protein 1(CLASP1)
1794	dedicator of cytokinesis 2(DOCK2)
23268	dynamin binding protein(DNMBP)
83450	dynein regulatory complex subunit 3(DRC3)
1605	dystroglycan 1(DAG1)
256364	echinoderm microtubule associated protein like 3(EML3)
56776	formin 2(FMN2)
9270	integrin subunit beta 1 binding protein 1(ITGB1BP1)
8100	intraflagellar transport 88(IFT88)
10300	katanin regulatory subunit B1(KATNB1)
11275	kelch like family member 2(KLHL2)
56992	kinesin family member 15(KIF15)
89953	kinesin light chain 4(KLC4)
9735	kinetochore associated 1(KNTC1)
9112	metastasis associated 1(MTA1)
4650	myosin IXB(MYO9B)
2908	nuclear receptor subfamily 3 group C member 1(NR3C1)
64098	parvin gamma(PARVG)
5493	periplakin(PPL)
26499	pleckstrin 2(PLEK2)
143	poly(ADP-ribose) polymerase family member 4(PARP4)
79834	pseudopodium enriched atypical kinase 1(PEAK1)
51115	regulator of microtubule dynamics 1(RMDN1)
25897	ring finger protein 19A, RBR E3 ubiquitin protein ligase(RNF19A)
6443	sarcoglycan beta(SGCB)
57477	shroom family member 4(SHROOM4)
22933	sirtuin 2(SIRT2)
6683	spastin(SPAST)
6712	spectrin beta, non-erythrocytic 2(SPTBN2)
79582	sperm associated antigen 16(SPAG16)
200162	sperm associated antigen 17(SPAG17)
25876	sperm flagellar 1(SPEF1)
2040	stomatin(STOM)
54221	syntrophin gamma 2(SNTG2)
55761	tetratricopeptide repeat domain 17(TTC17)
79809	tetratricopeptide repeat domain 21B(TTC21B)
23331	tetratricopeptide repeat domain 28(TTC28)
9321	thyroid hormone receptor interactor 11(TRIP11)
11011	tousled like kinase 2(TLK2)
91147	transmembrane protein 67(TMEM67)
7170	tropomyosin 3(TPM3)
10844	tubulin gamma complex associated protein 2(TUBGCP2)
23093	tubulin tyrosine ligase like 5(TTLL5)
23503	zinc finger FYVE-type containing 26(ZFYVE26)
51364	zinc finger MYND-type containing 10(ZMYND10)

mRNA splicing

ENTREZ_GENE_ID	GENE NAME
51202	DEAD-box helicase 47(DDX47)
11157	LSM6 homolog, U6 small nuclear RNA and mRNA degradation associated(LSM6)
51747	LUC7 like 3 pre-mRNA splicing factor(LUC7L3)
84991	RNA binding motif protein 17(RBM17)
58517	RNA binding motif protein 25(RBM25)
9169	SR-related CTD associated factor 11(SCAF11)
23517	Ski2 like RNA helicase 2(SKIV2L2)
79650	U6 snRNA biogenesis phosphodiesterase 1(USB1)
51729	WW domain binding protein 11(WBP11)
56949	XPA binding protein 2(XAB2)
22794	cancer susceptibility candidate 3(CASC3)
708	complement C1q binding protein(C1QBP)
51340	crooked neck pre-mRNA splicing factor 1(CRNKL1)
10150	muscleblind like splicing regulator 2(MBNL2)
55015	pre-mRNA processing factor 39(PRPF39)
24148	pre-mRNA processing factor 6(PRPF6)
11171	serine/threonine kinase receptor associated protein(STRAP)
9410	small nuclear ribonucleoprotein U5 subunit 40(SNRNP40)
10262	splicing factor 3b subunit 4(SF3B4)
9092	squamous cell carcinoma antigen recognized by T-cells 1(SART1)
8233	zinc finger CCCH-type, RNA binding motif and serine/arginine rich 2(ZRSR2)

Protein biosynthesis

ENTREZ_GENE_ID	GENE NAME
60558	GUF1 homolog, GTPase(GUF1)
6829	SPT5 homolog, DSIF elongation factor subunit(SUPT5H)
80325	ankyrin repeat and BTB domain containing 1(ABTB1)
57038	arginyl-tRNA synthetase 2, mitochondrial(RARS2)
55157	aspartyl-tRNA synthetase 2, mitochondrial(DARS2)
1615	aspartyl-tRNA synthetase(DARS)
8894	eukaryotic translation initiation factor 2 subunit beta(EIF2S2)
83939	eukaryotic translation initiation factor 2A(EIF2A)
1967	eukaryotic translation initiation factor 2B subunit alpha(EIF2B1)
8665	eukaryotic translation initiation factor 3 subunit F(EIF3F)
1975	eukaryotic translation initiation factor 4B(EIF4B)
1983	eukaryotic translation initiation factor 5(EIF5)
54516	mitochondrial translational release factor 1 like(MTRF1L)
2193	phenylalanyl-tRNA synthetase alpha subunit(FARSA)
25973	prolyl-tRNA synthetase 2, mitochondrial (putative)(PARS2)
7453	tryptophanyl-tRNA synthetase(WARS)

Ribosome biogenesis in eukaryotes

ENTREZ_GENE_ID	GENE NAME
22907	DExH-box helicase 30(DHX30)
51077	FCF1 rRNA-processing protein(FCF1)
55127	HEAT repeat containing 1(HEATR1)
387338	NOP2/Sun RNA methyltransferase family member 4(NSUN4)
51602	NOP58 ribonucleoprotein(NOP58)
708	complement C1q binding protein(C1QBP)
81875	interferon stimulated exonuclease gene 20 like 2(ISG20L2)
4931	nuclear VCP-like(NVL)
23212	ribosome biogenesis regulator homolog(RRS1)

rRNA processing

ENTREZ_GENE_ID	GENE NAME
51202	DEAD-box helicase 47(DDX47)
22894	DIS3 homolog, exosome endoribonuclease and 3'-5' exoribonuclease(DIS3)
51077	FCF1 rRNA-processing protein(FCF1)
55127	HEAT repeat containing 1(HEATR1)
11157	LSM6 homolog, U6 small nuclear RNA and mRNA degradation associated(LSM6)
387338	NOP2/Sun RNA methyltransferase family member 4(NSUN4)
23517	Ski2 like RNA helicase 2(SKIV2L2)
27340	UTP20, small subunit processome component(UTP20)
51729	WW domain binding protein 11(WBP11)
23016	exosome component 7(EXOSC7)
11340	exosome component 8(EXOSC8)
5393	exosome component 9(EXOSC9)
2091	fibrillarin(FBL)
25926	nucleolar protein 11(NOL11)

mRNA processing

ENTREZ_GENE_ID	GENE NAME
51202	DEAD-box helicase 47(DDX47)
22944	Kin17 DNA and RNA binding protein(KIN)
11157	LSM6 homolog, U6 small nuclear RNA and mRNA degradation associated(LSM6)
51747	LUC7 like 3 pre-mRNA splicing factor(LUC7L3)
84991	RNA binding motif protein 17(RBM17)
58517	RNA binding motif protein 25(RBM25)
9169	SR-related CTD associated factor 11(SCAF11)
23517	Ski2 like RNA helicase 2(SKIV2L2)
79650	U6 snRNA biogenesis phosphodiesterase 1(USB1)
51729	WW domain binding protein 11(WBP11)
56949	XPA binding protein 2(XAB2)
22794	cancer susceptibility candidate 3(CASC3)
55783	cap methyltransferase 2(CMTR2)
10978	cleavage and polyadenylation factor I subunit 1(CLP1)
29894	cleavage and polyadenylation specific factor 1(CPSF1)
708	complement C1q binding protein(C1QBP)
51340	crooked neck pre-mRNA splicing factor 1(CRNKL1)
10150	muscleblind like splicing regulator 2(MBNL2)
79810	pentatricopeptide repeat domain 2(PTCD2)
55015	pre-mRNA processing factor 39(PRPF39)
24148	pre-mRNA processing factor 6(PRPF6)
11171	serine/threonine kinase receptor associated protein(STRAP)
9410	small nuclear ribonucleoprotein U5 subunit 40(SNRNP40)
10262	splicing factor 3b subunit 4(SF3B4)
9092	squamous cell carcinoma antigen recognized by T-cells 1(SART1)
7884	stem-loop binding protein(SLBP)
8233	zinc finger CCCH-type, RNA binding motif and serine/arginine rich 2(ZRSR2)

tRNA processing

ENTREZ_GENE_ID	GENE NAME
24140	FtsJ RNA methyltransferase homolog 1 (E. coli)(FTSJ1)
11157	LSM6 homolog, U6 small nuclear RNA and mRNA degradation associated(LSM6)
51493	RNA 2',3'-cyclic phosphate and 5'-OH ligase(RTCB)
10978	cleavage and polyadenylation factor I subunit 1(CLP1)
25821	mitochondrial tRNA translation optimization 1(MTO1)
150962	pseudouridylate synthase 10(PUS10)
54802	tRNA isopentenyltransferase 1(TRIT1)
55621	tRNA methyltransferase 1(TRMT1)
54482	tRNA methyltransferase 13 homolog(TRMT13)
152992	tRNA methyltransferase 44 homolog (S. cerevisiae)(TRMT44)
55253	tRNA-yW synthesizing protein 1 homolog(TYW1)
129450	tRNA-yW synthesizing protein 5(TYW5)

RNA binding

ENTREZ_GENE_ID	GENE NAME
79026	AHNAK nucleoprotein(AHNAK)
23	ATP binding cassette subfamily F member 1(ABCF1)
84811	BUD13 homolog(BUD13)
22794	CASC3 exon junction complex subunit(CASC3)
51202	DEAD-box helicase 47(DDX47)
54555	DEAD-box helicase 49(DDX49)

mitochondrion

ENTREZ_GENE_ID	GENE NAME
3155	3-hydroxy-3-methylglutaryl-CoA lyase(HMGCL)
622	3-hydroxybutyrate dehydrogenase 1(BDH1)
18	4-aminobutyrate aminotransferase(ABAT)
112817	4-hydroxy-2-oxoglutarate aldolase 1(HOGA1)
598	BCL2 like 1(BCL2L1)
284106	CDGSH iron sulfur domain 3(CISD3)
22907	DExH-box helicase 30(DHX30)
3980	DNA ligase 3(LIG3)
3301	DnaJ heat shock protein family (Hsp40) member A1(DNAJA1)
2874	G protein pathway suppressor 2(GPS2)
60558	GTP binding elongation factor GUF1(GUF1)
55127	HEAT repeat containing 1(HEATR1)
138151	NACC family member 2(NACC2)
4719	NADH:ubiquinone oxidoreductase core subunit S1(NDUFS1)
60491	NGG1 interacting factor 3 like 1(NIF3L1)
10201	NME/NM23 nucleoside diphosphate kinase 6(NME6)
80224	NUBP iron-sulfur cluster assembly factor, mitochondrial(NUBPL)
4976	OPA1 mitochondrial dynamin like GTPase(OPA1)
5888	RAD51 recombinase(RAD51)
5903	RAN binding protein 2(RANBP2)
25996	RNA exonuclease 2(REXO2)
5442	RNA polymerase mitochondrial(POLRMT)
84881	RNA pseudouridine synthase D4(RPUSD4)
57154	SMAD specific E3 ubiquitin protein ligase 1(SMURF1)
6687	SPG7 matrix AAA peptidase subunit, paraplegin(SPG7)
38	acetyl-CoA acetyltransferase 1(ACAT1)
10449	acetyl-CoA acyltransferase 2(ACAA2)
32	acetyl-CoA carboxylase beta(ACACB)
48	aconitase 1(ACO1)
36	acyl-CoA dehydrogenase short/branched chain(ACADSB)
79611	acyl-CoA synthetase short chain family member 3(ACSS3)
160428	aldehyde dehydrogenase 1 family member L2(ALDH1L2)
8846	alkB homolog 1, histone H2A dioxygenase(ALKBH1)
221120	alkB homolog 3, alpha-ketoglutarate dependent dioxygenase(ALKBH3)
57038	arginyl-tRNA synthetase 2, mitochondrial(RARS2)
55157	aspartyl-tRNA synthetase 2, mitochondrial(DARS2)
10902	bromodomain containing 8(BRD8)
8192	caseinolytic mitochondrial matrix peptidase proteolytic subunit(CLPP)
841	caspase 8(CASP8)
10087	ceramide transporter 1(CERT1)
728568	chromosome 12 open reading frame 73(C12orf73)
708	complement C1q binding protein(C1QBP)
1537	cytochrome c1(CYC1)
5447	cytochrome p450 oxidoreductase(POR)
55101	distal membrane arm assembly component 2(DMAC2)
1892	enoyl-CoA hydratase, short chain 1(ECHS1)
55218	exonuclease 3'-5' domain containing 2(EXD2)
3607	forkhead box K2(FOXK2)
2954	glutathione S-transferase zeta 1(GSTZ1)
3313	heat shock protein family A (Hsp70) member 9(HSPA9)
8520	histone acetyltransferase 1(HAT1)
3416	insulin degrading enzyme(IDE)
64135	interferon induced with helicase C domain 1(IFIH1)
3658	iron responsive element binding protein 2(IREB2)
122961	iron-sulfur cluster assembly 2(ISCA2)
3419	isocitrate dehydrogenase (NAD(+)) 3 catalytic subunit alpha(IDH3A)
10128	leucine rich pentatricopeptide repeat containing(LRPPRC)
8513	lipase F, gastric type(LIPF)
64745	methyltransferase like 17(METTL17)
23788	mitochondrial carrier 2(MTCH2)
56181	mitochondrial fission regulator 1 like(MTFR1L)
64928	mitochondrial ribosomal protein L14(MRPL14)
54948	mitochondrial ribosomal protein L16(MRPL16)
6150	mitochondrial ribosomal protein L23(MRPL23)
51263	mitochondrial ribosomal protein L30(MRPL30)
55168	mitochondrial ribosomal protein S18A(MRPS18A)
51116	mitochondrial ribosomal protein S2(MRPS2)
64951	mitochondrial ribosomal protein S24(MRPS24)
64432	mitochondrial ribosomal protein S25(MRPS25)
64949	mitochondrial ribosomal protein S26(MRPS26)
25821	mitochondrial tRNA translation optimization 1(MTO1)
51102	mitochondrial trans-2-enoyl-CoA reductase(MECR)
54516	mitochondrial translation release factor 1 like(MTRF1L)
25983	neuroguidin(NGDN)
55335	nipsnap homolog 3B(NIPSNAP3B)
2908	nuclear receptor subfamily 3 group C member 1(NR3C1)
79810	pentatricopeptide repeat domain 2(PTCD2)
23761	phosphatidylserine decarboxylase(PISD)
23241	phosphofurin acidic cluster sorting protein 2(PACS2)
5264	phytanoyl-CoA 2-hydroxylase(PHYH)
25973	prolyl-tRNA synthetase 2, mitochondrial(PARS2)
150962	pseudouridine synthase 10(PUS10)
10928	ralA binding protein 1(RALBP1)
55288	ras homolog family member T1(RHOT1)
84947	serine active site containing 1(SERAC1)
6472	serine hydroxymethyltransferase 2(SHMT2)
22933	sirtuin 2(SIRT2)
84275	solute carrier family 25 member 33(SLC25A33)
51629	solute carrier family 25 member 39(SLC25A39)
132671	spermatogenesis associated 18(SPATA18)
6770	steroidogenic acute regulatory protein(STAR)
2040	stomatin(STOM)
6389	succinate dehydrogenase complex flavoprotein subunit A(SDHA)
58472	sulfide quinone oxidoreductase(SQOR)
8773	synaptosome associated protein 23(SNAP23)
54802	tRNA isopentenyltransferase 1(TRIT1)
54881	testis expressed 10(TEX10)
7019	transcription factor A, mitochondrial(TFAM)
9238	transforming growth factor beta regulator 4(TBRG4)
54968	transmembrane protein 70(TMEM70)

56919	DEAH-box helicase 33(DHX33)
22907	DExH-box helicase 30(DHX30)
2079	ERH mRNA splicing and mitosis factor(ERH)
51077	FCF1 rRNA-processing protein(FCF1)
55127	HEAT repeat containing 1(HEATR1)
84298	LLP homolog, long-term synaptic facilitation factor(LLPH)
11157	LSM6 homolog, U6 small nuclear RNA and mRNA degradation associated(LSM6)
51747	LUC7 like 3 pre-mRNA splicing factor(LUC7L3)
23517	Mtr4 exosome RNA helicase(MTREX)
51602	NOP58 ribonucleoprotein(NOP58)
161424	NOP9 nucleolar protein(NOP9)
5822	PWP2 small subunit processome component(PWP2)
51493	RNA 2',3'-cyclic phosphate and 5'-OH ligase(RTCB)
58517	RNA binding motif protein 25(RBM25)
155435	RNA binding motif protein 33(RBM33)
5431	RNA polymerase II subunit B(POLR2B)
5442	RNA polymerase mitochondrial(POLRMT)
84881	RNA pseudouridine synthase D4(RPUSD4)
23168	RTF1 homolog, Paf1/RNA polymerase II complex component(RTF1)
11196	SEC23 interacting protein(SEC23IP)
6829	SPT5 homolog, DSIF elongation factor subunit(SUPT5H)
9169	SR-related CTD associated factor 11(SCAF11)
6734	SRP receptor subunit alpha(SRPRA)
5976	UPF1 RNA helicase and ATPase(UPF1)
8615	USO1 vesicle transport factor(USO1)
27340	UTP20 small subunit processome component(UTP20)
51729	WW domain binding protein 11(WBP11)
10449	acetyl-CoA acyltransferase 2(ACAA2)
81	actinin alpha 4(ACTN4)
54882	ankyrin repeat and KH domain containing 1(ANKHD1)
57038	arginyl-tRNA synthetase 2, mitochondrial(RARS2)
1615	aspartyl-tRNA synthetase 1(DARS1)
1454	casein kinase 1 epsilon(CSNK1E)
10575	chaperonin containing TCP1 subunit 4(CCT4)
255101	cilia and flagella associated protein 65(CFAP65)
26093	coiled-coil domain containing 9(CCDC9)
51340	crooked neck pre-mRNA splicing factor 1(CRNKL1)
8894	eukaryotic translation initiation factor 2 subunit beta(EIF2S2)
1975	eukaryotic translation initiation factor 4B(EIF4B)
1983	eukaryotic translation initiation factor 5(EIF5)
5393	exosome component 9(EXOSC9)
2091	fibrillarin(FBL)
2804	golgin B1(GOLGB1)
3313	heat shock protein family A (Hsp70) member 9(HSPA9)
81875	interferon stimulated exonuclease gene 20 like 2(ISG20L2)
10128	leucine rich pentatricopeptide repeat containing(LRPPRC)
64928	mitochondrial ribosomal protein L14(MRPL14)
64951	mitochondrial ribosomal protein S24(MRPS24)
64949	mitochondrial ribosomal protein S26(MRPS26)
25821	mitochondrial tRNA translation optimization 1(MTO1)
10150	muscleblind like splicing regulator 2(MBNL2)
25983	neuroguidin(NGDN)
51070	nitric oxide synthase interacting protein(NOSIP)
4931	nuclear VCP like(NVL)
25926	nucleolar protein 11(NOL11)
4676	nucleosome assembly protein 1 like 4(NAP1L4)
79810	pentatricopeptide repeat domain 2(PTCD2)
9360	peptidylprolyl isomerase G(PPIG)
2193	phenylalanyl-tRNA synthetase subunit alpha(FARSA)
8761	poly(A) binding protein cytoplasmic 4(PABPC4)
5073	poly(A)-specific ribonuclease(PARN)
24148	pre-mRNA processing factor 6(PRPF6)
27341	ribosomal RNA processing 7 homolog A(RRP7A)
23212	ribosome biogenesis regulator 1 homolog(RRS1)
11171	serine/threonine kinase receptor associated protein(STRAP)
6729	signal recognition particle 54(SRP54)
6731	signal recognition particle 72(SRP72)
9410	small nuclear ribonucleoprotein U5 subunit 40(SNRNP40)
9092	spliceosome associated factor 1, recruiter of U4/U6.U5 tri-snRNP(SART1)
10262	splicing factor 3b subunit 4(SF3B4)
7884	stem-loop binding protein(SLBP)
55621	tRNA methyltransferase 1(TRMT1)
7019	transcription factor A, mitochondrial(TFAM)
26608	transducin beta like 2(TBL2)
9238	transforming growth factor beta regulator 4(TBRG4)
7531	tyrosine 3-monooxygenase/tryptophan 5-monooxygenase activation protein epsilon(Y
57602	ubiquitin specific peptidase 36(USP36)
85451	unk zinc finger(UNK)
7415	valosin containing protein(VCP)
124245	zinc finger CCCH-type containing 18(ZC3H18)
90441	zinc finger protein 622(ZNF622)

84300	ubiquinol-cytochrome c reductase complex assembly factor 2(UQCC2)
7384	ubiquinol-cytochrome c reductase core protein 1(UQCRC1)
7350	uncoupling protein 1(UCP1)
7351	uncoupling protein 2(UCP2)
53349	zinc finger FYVE-type containing 1(ZFYVE1)
