# Supplementary material for: Transcriptomes of Injured Lamprey Axon Tips: Single-Cell RNA-Seq Suggests Differential Involvement of MAPK Signaling Pathways in Axon Retraction and Regeneration after Spinal Cord Injury
Source: Cells. 2022 Jul 27;11(15):2320. doi: 10.3390/cells11152320 (PMC9367414; doi:10.3390/cells11152320)
Supplement: Supplementary file 1 [file cells-11-02320-s001.zip › cells-1803217-supplementary/cells-1803217supplementary/cells-1803217-figures and tables/Table S6.pdf]

A

	R^2
G1,2	0.107
G1,3	0.06991
G1,4	0.05388
G1,5	0.1314
G1,6	0.1571
G1,7	0.1283
G1,8	0.07303
G1,9	0.103
G1,10	0.1271
G2,3	0.05715
G2,4	0.05608
G2,5	0.1014
G2,6	0.1278
G2,7	0.1096
G2,8	0.06505
G2,9	0.1054
G2,10	0.0961
G3,4	0.06769
G3,5	0.05311
G3,6	0.1025
G3,7	0.06747
G3,8	0.1167
G3,9	0.01841
G3,10	0.0723
G4,5	0.04496
G4,6	0.06615
G4,7	0.05101
G4,8	0.07806
G4,9	0.02391
G4,10	0.05287
G5,6	0.3652
G5,7	0.2447
G5,8	0.047
G5,9	0.1947
G5,10	0.2359
G6,7	0.3291
G6,8	0.07468
G6,9	0.2068
G6,10	0.3497
G7,8	0.06409
G7,9	0.1785
G7,10	0.2109
G8,9	0.01463
G8,10	0.06232
G9,10	0.1401
S1,2	0.01909
S1,3	0.1477
S1,4	0.04939
S1,5	0.1695
S1,6	0.1504
S1,7	0.1805
S1,8	0.1626
S1,9	0.1518
S2,3	0.01145
S2,4	0.07875
S2,5	0.01149
S2,6	0.02989
S2,7	0.01603
S2,8	0.01791
S2,9	0.02477
S3,4	0.04105
S3,5	0.2064
S3,6	0.1627
S3,7	0.2165
S3,8	0.2033
S3,9	0.163
S4,5	0.04712
S4,6	0.08563
S4,7	0.05958
S4,8	0.0637
S4,9	0.05937
S5,6	0.3155
S5,7	0.4376
S5,8	0.3871
S5,9	0.2792
S6,7	0.3282
S6,8	0.2965
S6,9	0.2213
S7,8	0.4285
S7,9	0.2522
S8,9	0.2383
R1,2	0.0368
R1,3	0.02979
R1,4	0.02511
R1,5	0.04384
R2,3	0.1872
R2,4	0.03849
R2,5	0.166
R3,4	0.0196
R3,5	0.1009
R4,5	0.04869

B

	G1	G2	G3	G4	G5	G6	G7	G8	G9	G10	sum-R^2
**G1**	1	0.107	0.06991	0.05388	0.1314	0.1571	0.1283	0.07303	0.103	0.1271	0.951
**G2**	0.107	1	0.05715	0.05608	0.1014	0.1278	0.1096	0.06505	0.1054	0.0961	0.826
**G3**	0.06991	0.05715	1	0.06769	0.05311	0.1025	0.06747	0.1167	0.01841	0.0723	0.625
**G4**	0.05388	0.05608	0.06769	1	0.04496	0.06615	0.05101	0.07806	0.02391	0.05287	0.495
**G5**	0.1314	0.1014	0.05311	0.04496	1	0.3652	0.2447	0.047	0.1947	0.2359	1.418
**G6**	0.1571	0.1278	0.1025	0.06615	0.3652	1	0.3291	0.07468	0.2068	0.3497	1.779
**G7**	0.1283	0.1096	0.06747	0.05101	0.2447	0.3291	1	0.06409	0.1785	0.2109	1.384
**G8**	0.07303	0.06505	0.1167	0.07806	0.047	0.07468	0.06409	1	0.01463	0.06232	0.596
**G9**	0.103	0.1054	0.01841	0.02391	0.1947	0.2068	0.1785	0.01463	1	0.1401	0.985
**G10**	0.1271	0.0961	0.0723	0.05287	0.2359	0.3497	0.2109	0.06232	0.1401	1	1.347

	S1	S2	S3	S4	S5	S6	S7	S8	S9	
**S1**	1	0.01909	0.1477	0.04939	0.1695	0.1504	0.1805	0.1626	0.1518	1.031
**S2**	0.01909	1	0.01145	0.07875	0.01149	0.02989	0.01603	0.01791	0.02477	0.209
**S3**	0.1477	0.01145	1	0.04105	0.2064	0.1627	0.2165	0.2033	0.163	1.152
**S4**	0.04939	0.07875	0.04105	1	0.04712	0.08563	0.05958	0.0637	0.05937	0.485
**S5**	0.1695	0.01149	0.2064	0.04712	1	0.3155	0.4376	0.3871	0.2792	1.854
**S6**	0.1504	0.02989	0.1627	0.08563	0.3155	1	0.3282	0.2965	0.2213	1.590
**S7**	0.1805	0.01603	0.2165	0.05958	0.4376	0.3282	1	0.4285	0.2522	1.919
**S8**	0.1626	0.01791	0.2033	0.0637	0.3871	0.2965	0.4285	1	0.2383	1.798
**S9**	0.1518	0.02477	0.163	0.05937	0.2792	0.2213	0.2522	0.2383	1	1.390

	R1	R2	R3	R4	R5	
**R1**	1	0.0368	0.02979	0.02511	0.04384	0.136
**R2**	0.0368	1	0.1872	0.03849	0.166	0.428
**R3**	0.02979	0.1872	1	0.0196	0.1009	0.337
**R4**	0.02511	0.03849	0.0196	1	0.04869	0.132
**R5**	0.04384	0.166	0.1009	0.04869	1	0.359
