# Supplementary material for: Transcriptomes of Injured Lamprey Axon Tips: Single-Cell RNA-Seq Suggests Differential Involvement of MAPK Signaling Pathways in Axon Retraction and Regeneration after Spinal Cord Injury
Source: Cells. 2022 Jul 27;11(15):2320. doi: 10.3390/cells11152320 (PMC9367414; doi:10.3390/cells11152320)
Supplement: Supplementary file 1 [file cells-11-02320-s001.zip › cells-1803217-supplementary/cells-1803217supplementary/cells-1803217-figures and tables/Table S7.pdf]

**map3k2-network**

| gene_ID | gene_symbol | Average (G) | Average (S) | Average(R) |
|---|---|---|---|---|
| ENSPMAG00000006946 | acox3 | 6.60 | 19.78 | 0.00 |
| ENSPMAG00000004296 | FAM118A | 3.60 | 21.67 | 26.80 |
| ENSPMAG00000006728 | fam208ab | 6.90 | 7.00 | 0.00 |
| ENSPMAG00000006084 | hhipl1 | 6.60 | 28.22 | 0.00 |
| ENSPMAG00000004255 | map2k2a | 17.30 | 73.44 | 0.00 |
| ENSPMAG00000005963 | map2k4a | 681.70 | 96.56 | 340.60 |
| ENSPMAG00000004782 | map2k6 | 14.10 | 105.89 | 18.20 |
| ENSPMAG00000008753 | map2k7 | 12.30 | 4.56 | 0.00 |
| ENSPMAG00000005783 | map3k1 | 9.10 | 11.22 | 0.00 |
| ENSPMAG00000007593 | MAP3K11 (1 of 2) | 506.60 | 61.44 | 0.00 |
| ENSPMAG00000000733 | MAP3K2 | 259.10 | 0.89 | 0.00 |
| ENSPMAG00000000044 | map3k4 | 18.00 | 0.00 | 18.60 |
| ENSPMAG00000005510 | map3k7 | 5.40 | 2.11 | 3.60 |
| ENSPMAG00000008402 | mapk12b | 12.80 | 16.56 | 32.00 |
| ENSPMAG00000001908 | MAPK14 (1 of 2) | 226.60 | 16.56 | 0.20 |
| ENSPMAG00000008420 | MAPK14 (2 of 2) | 6.30 | 3.89 | 18.60 |
| ENSPMAG00000000656 | MAPK8 (1 of 2) | 9.10 | 186.11 | 2.40 |
| ENSPMAG00000003756 | MAPK8 (2 of 2) | 4.20 | 18.56 | 232.80 |
| ENSPMAG00000000046 | nr2f1b | 0.30 | 20.33 | 0.00 |
| ENSPMAG00000004679 | ptprga | 44.90 | 0.22 | 0.20 |
| ENSPMAG00000005071 | smurf1 | 225.70 | 42.89 | 0.00 |
| ENSPMAG00000009839 | smyd3 (2 of 2) | 0.00 | 0.00 | 17.20 |
| ENSPMAG00000000902 | stk38b | 183.10 | 2.11 | 57.60 |
| ENSPMAG00000008800 | zfpm2b | 62.80 | 3.89 | 0.00 |
| ENSPMAG00000009252 | mapk1 | 1146.90 | 316.67 | 2033.20 |

**csnk1e-network**

| gene_ID | gene_symbol | Average (G) | Average (S) | Average(R) |
|---|---|---|---|---|
| ENSPMAG00000004171 | arntl1a | 1.00 | 0.67 | 0.00 |
| ENSPMAG00000003684 | bhlhe40 (1 of 2) | 218.60 | 19.56 | 0.00 |
| ENSPMAG00000006788 | bhlhe41 | 24.90 | 12.22 | 0.00 |
| ENSPMAG00000002704 | clock3 | 156.80 | 9.78 | 287.20 |
| ENSPMAG00000004074 | cry1b | 35.20 | 0.89 | 0.00 |
| ENSPMAG00000001522 | csnk1a1 | 163.20 | 1.11 | 0.00 |
| ENSPMAG00000007664 | csnk1e | 214.20 | 1.11 | 0.00 |
| ENSPMAG00000006413 | dennd4a | 5.50 | 50.56 | 5.20 |
| ENSPMAG00000007929 | esyt3 | 430.00 | 79.11 | 3.40 |
| ENSPMAG00000006961 | gstcd | 1.00 | 4.22 | 0.00 |
| ENSPMAG00000003750 | jkamp | 20.70 | 230.56 | 21.80 |
| ENSPMAG00000002322 | lrrc14b | 16.70 | 0.44 | 0.00 |
| ENSPMAG00000004099 | memo1 | 663.50 | 107.78 | 178.40 |
| ENSPMAG00000008658 | mtnr1ba | 0.00 | 0.00 | 0.40 |
| ENSPMAG00000001830 | mtss1l | 40.00 | 63.11 | 118.80 |
| ENSPMAG00000007632 | NPAS3 (1 of 2) | 11.30 | 10.33 | 0.00 |
| ENSPMAG00000008468 | NR1D1 (2 of 2) | 11.60 | 0.33 | 0.00 |
| ENSPMAG00000003707 | phf20a | 243.40 | 1.78 | 229.60 |
| ENSPMAG00000009524 | ppp1r12a | 94.00 | 72.89 | 25.00 |
| ENSPMAG00000004996 | tcp11l2 | 226.90 | 7.11 | 0.00 |
| ENSPMAG00000008991 | timeless | 11.30 | 18.56 | 0.20 |
| ENSPMAG00000007315 | tipin | 125.90 | 381.56 | 0.00 |
| ENSPMAG00000001539 | tmem161 | 3.00 | 7.56 | 0.00 |
