# Supplementary figures and images for: Transcriptomes of Injured Lamprey Axon Tips: Single-Cell RNA-Seq Suggests Differential Involvement of MAPK Signaling Pathways in Axon Retraction and Regeneration after Spinal Cord Injury
Source: Cells. 2022 Jul 27;11(15):2320. doi: 10.3390/cells11152320 (PMC9367414; doi:10.3390/cells11152320)

# Growing tips (10)

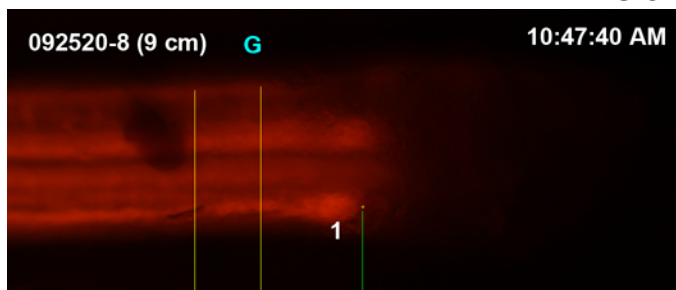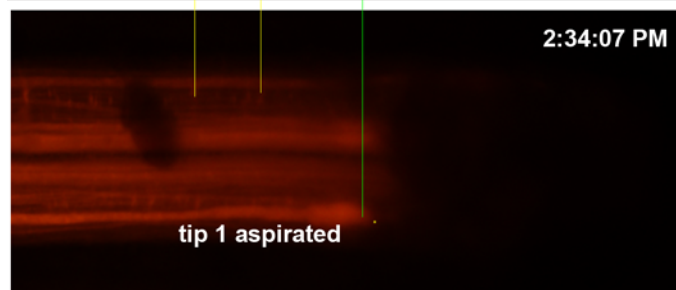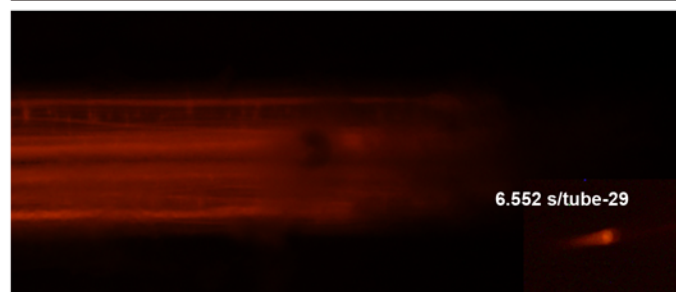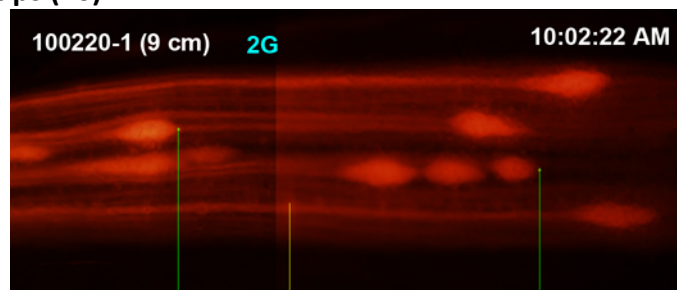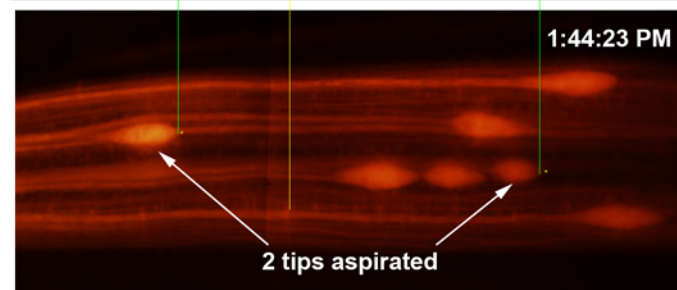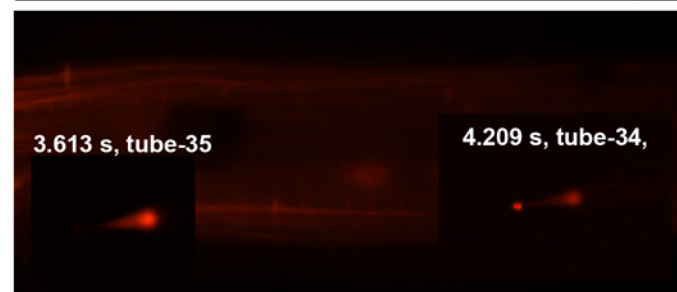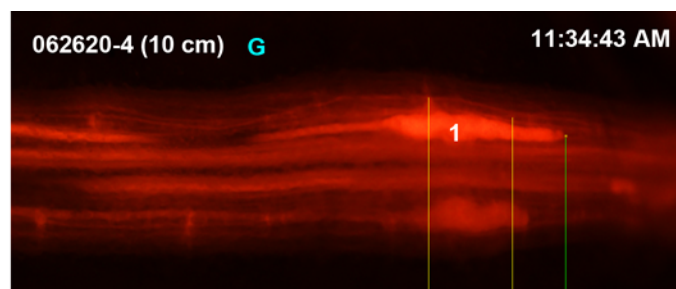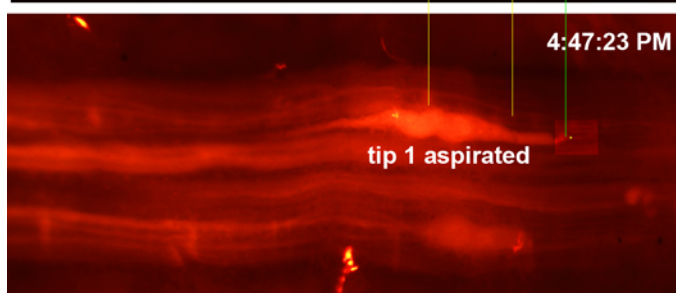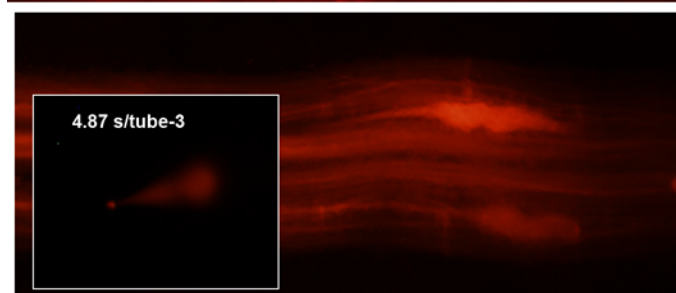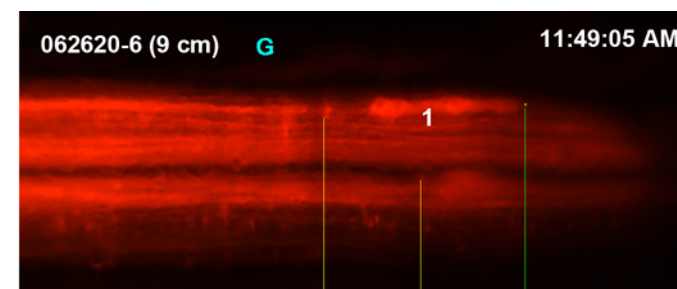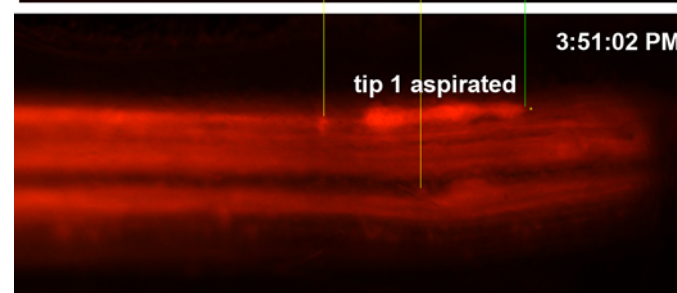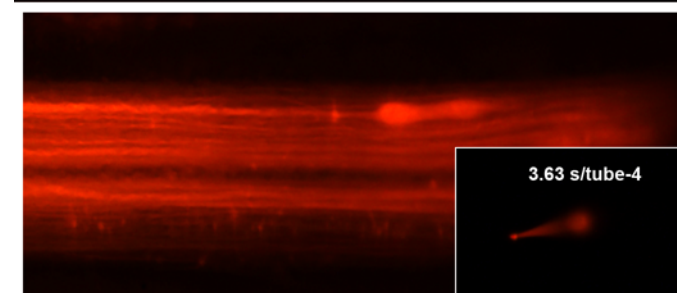

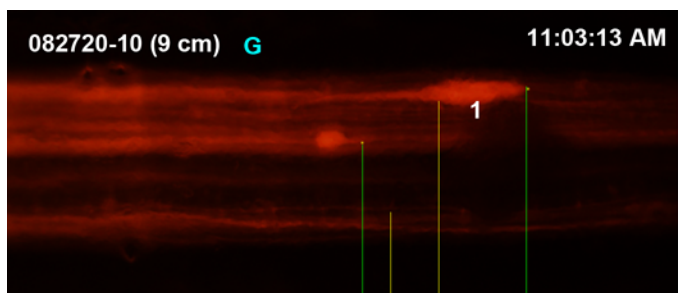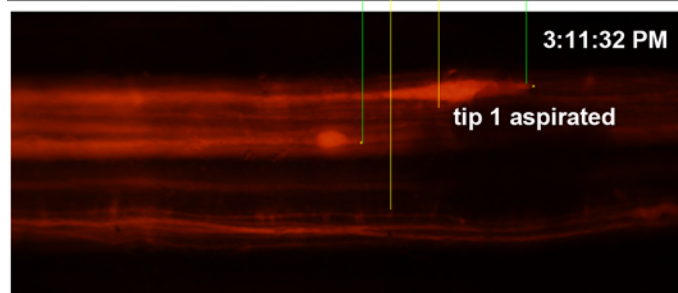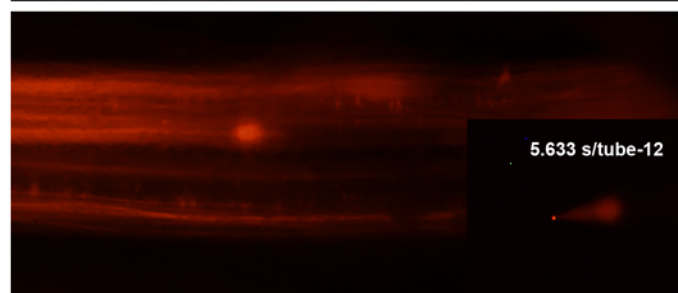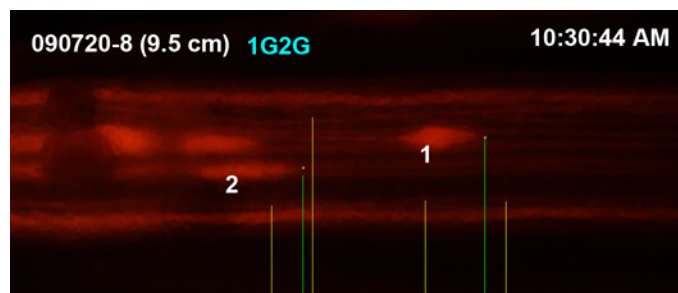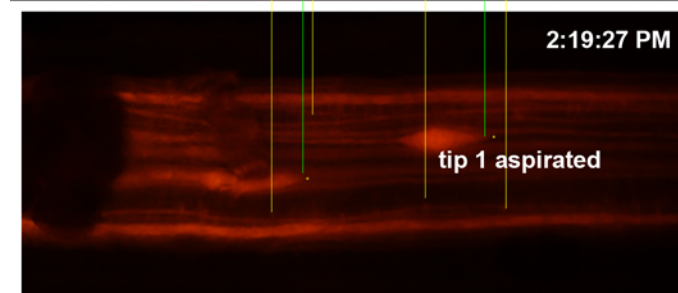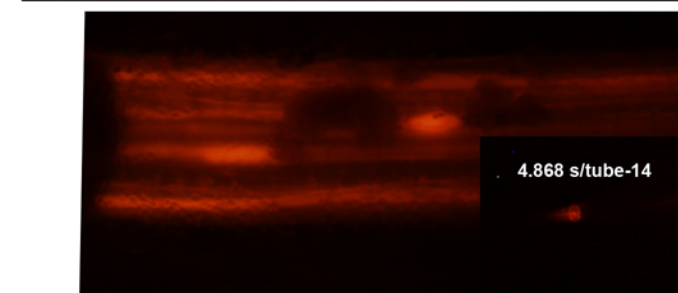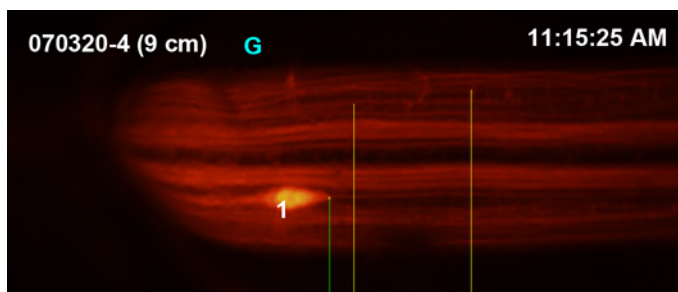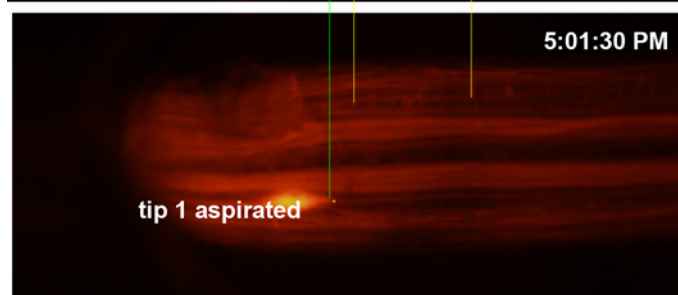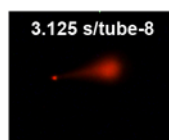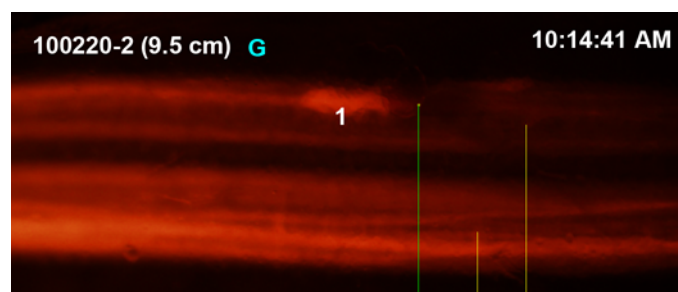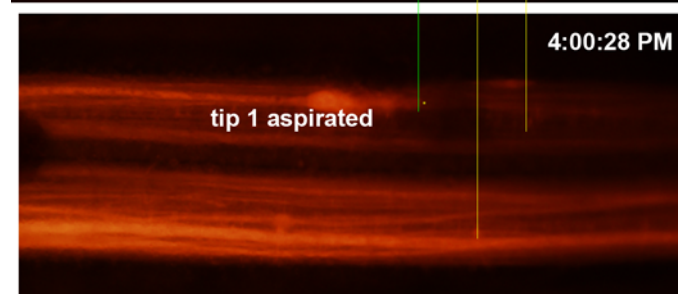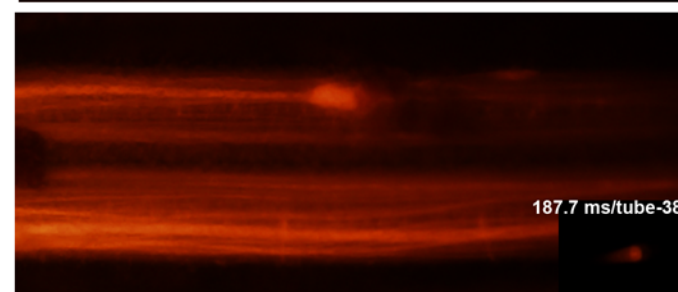

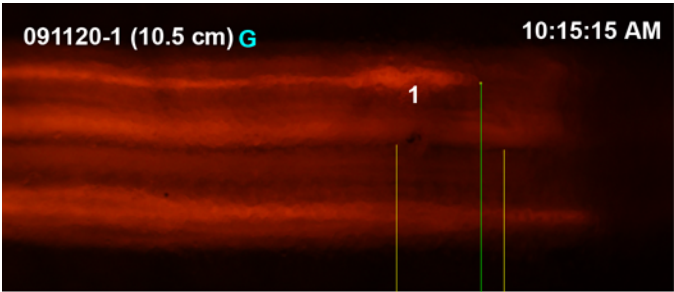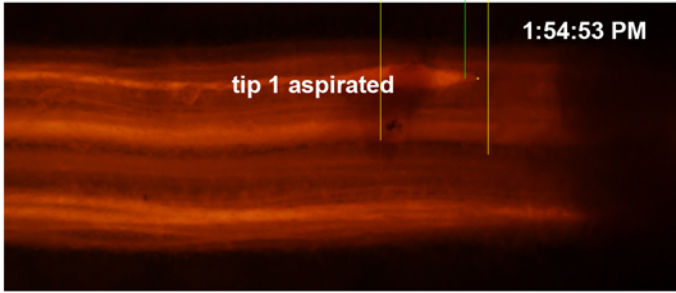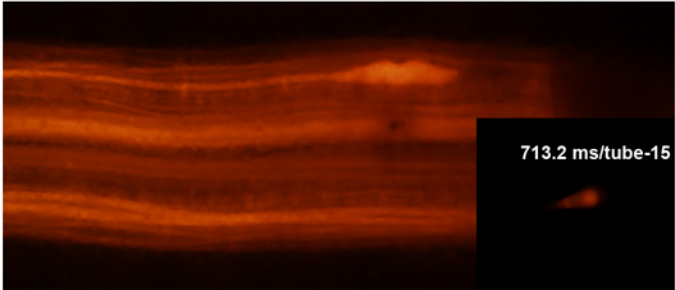

Static tips (9)

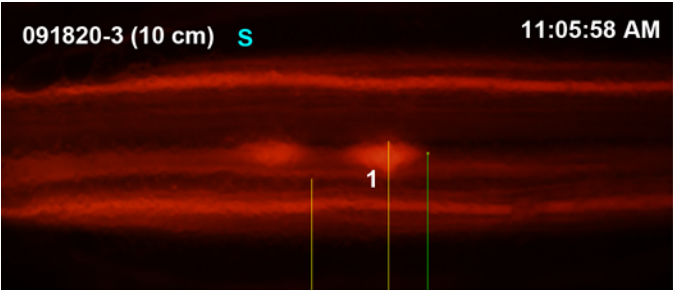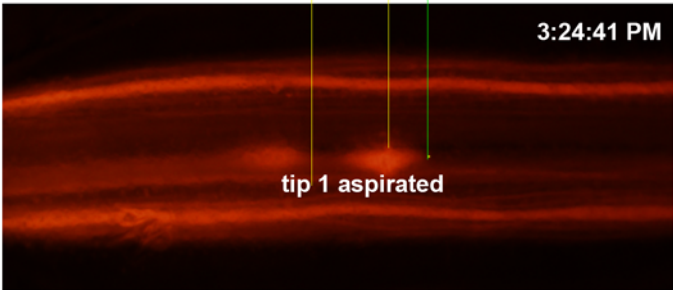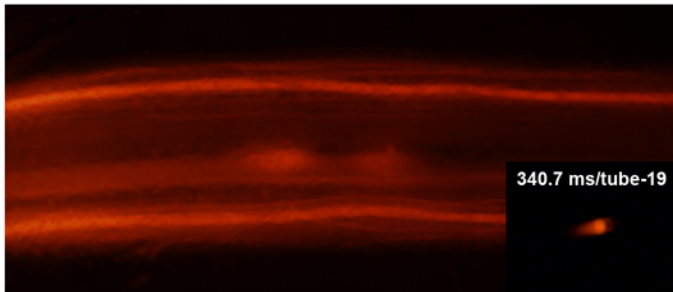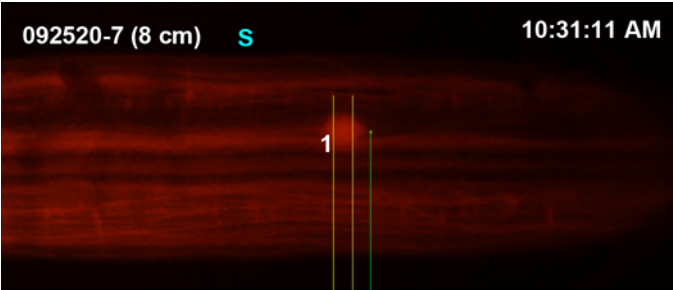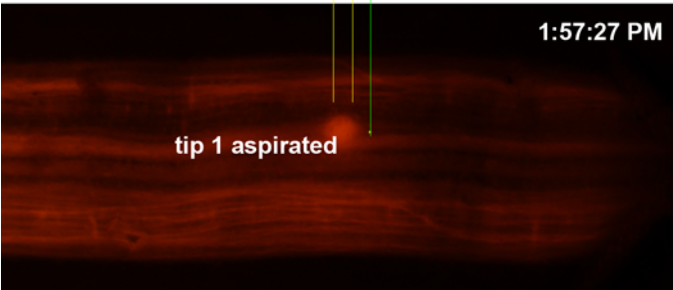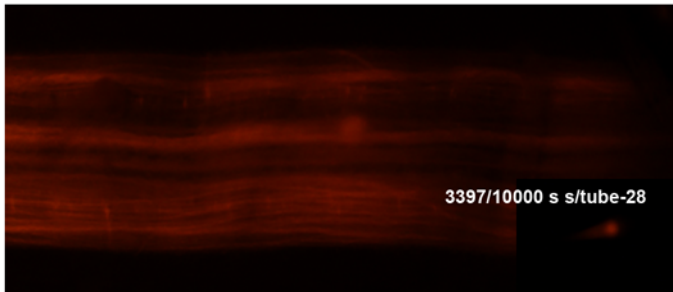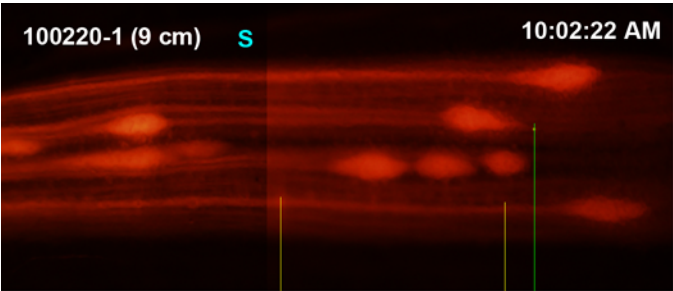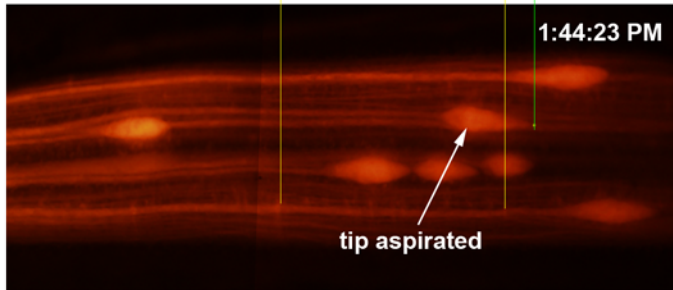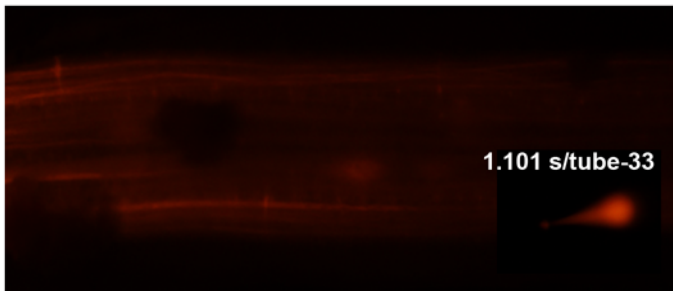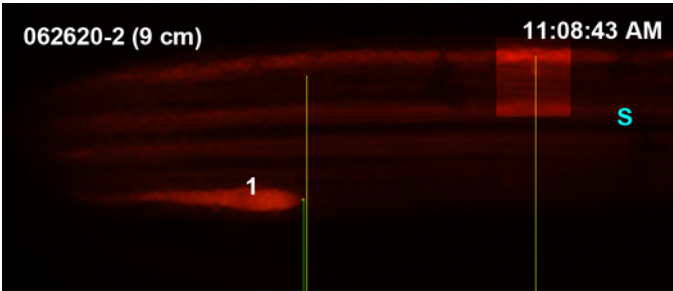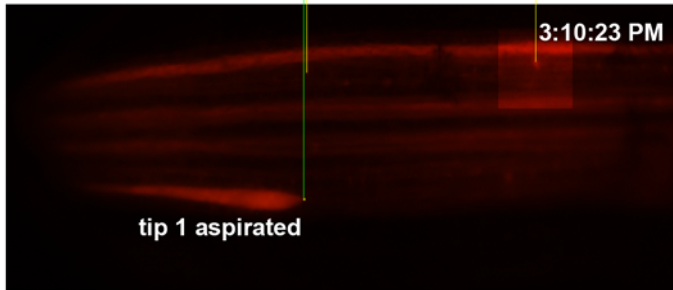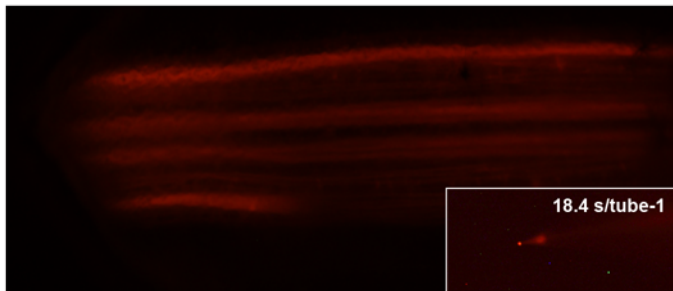

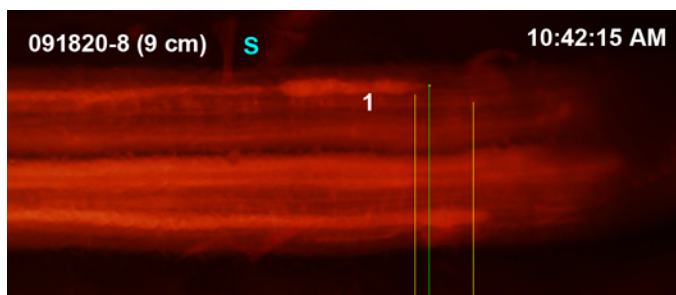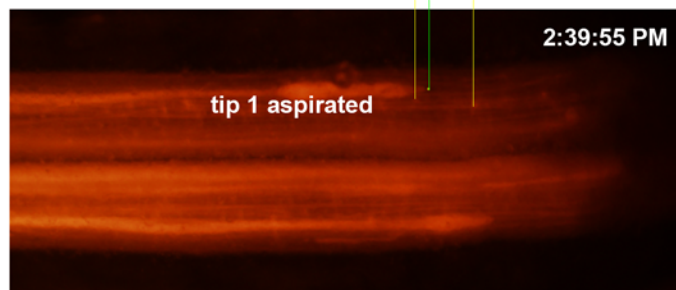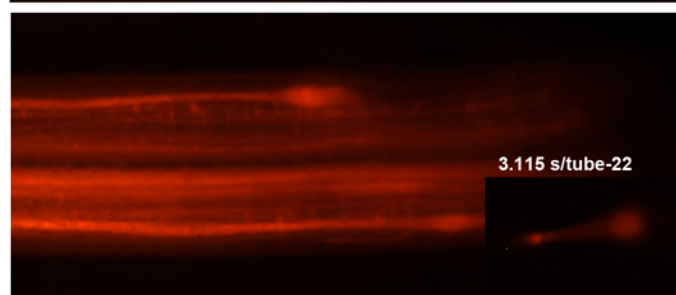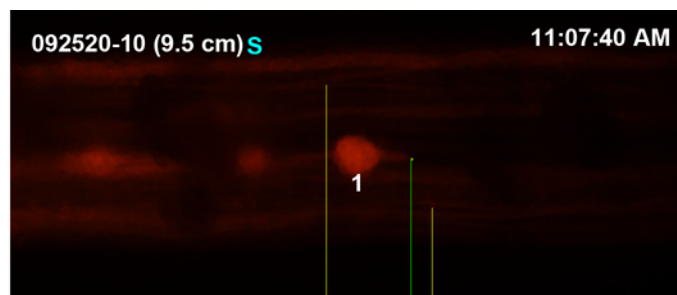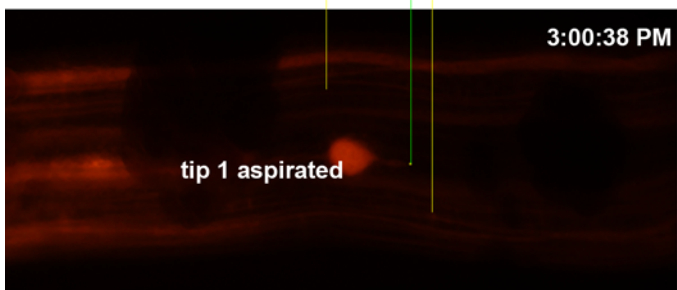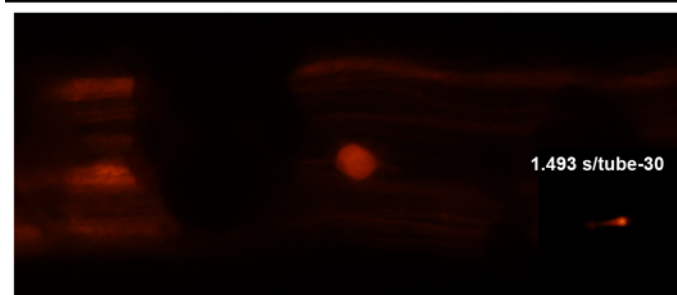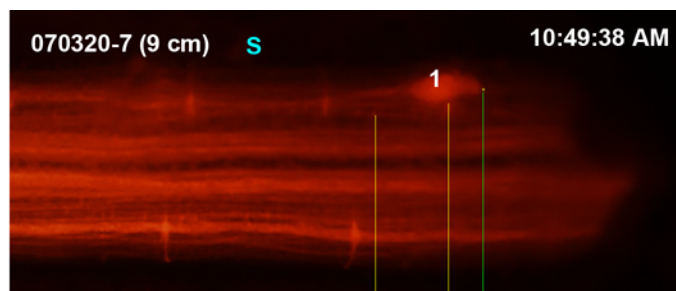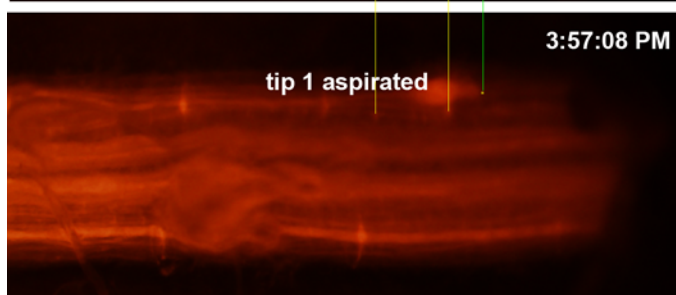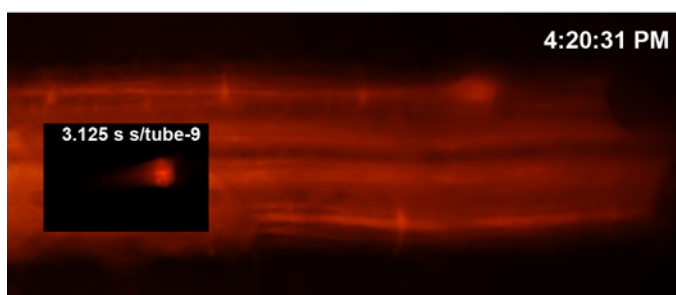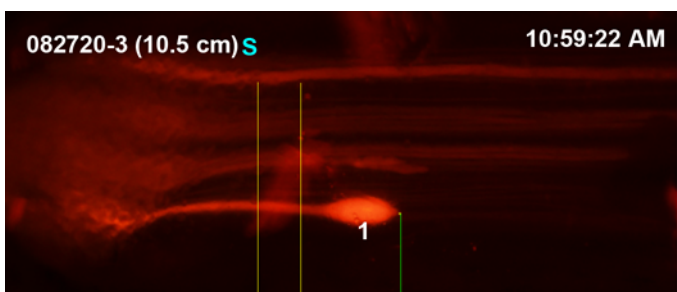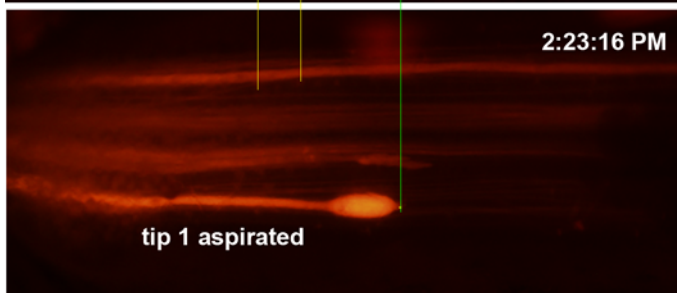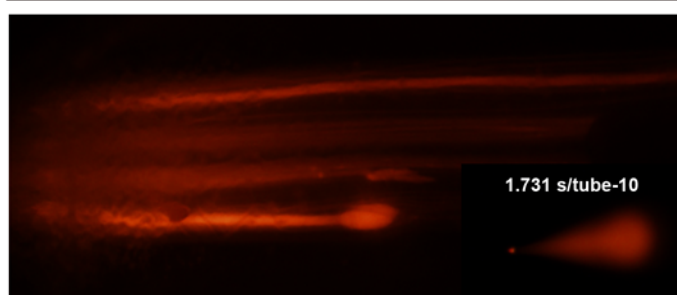

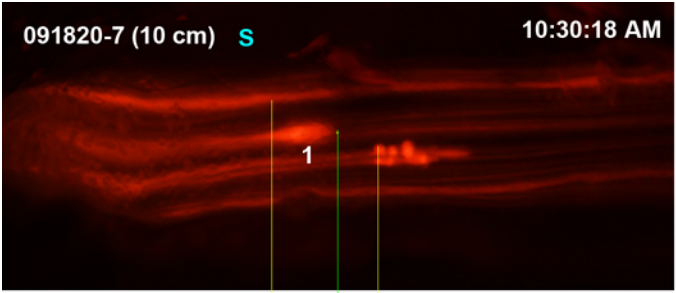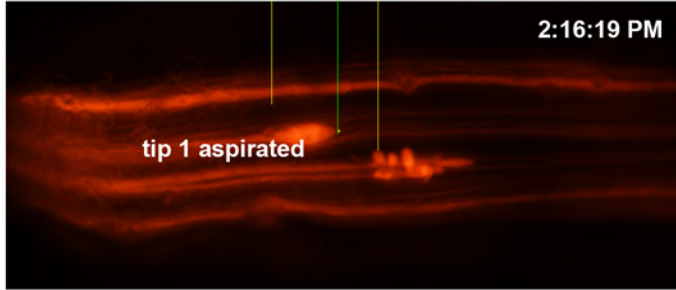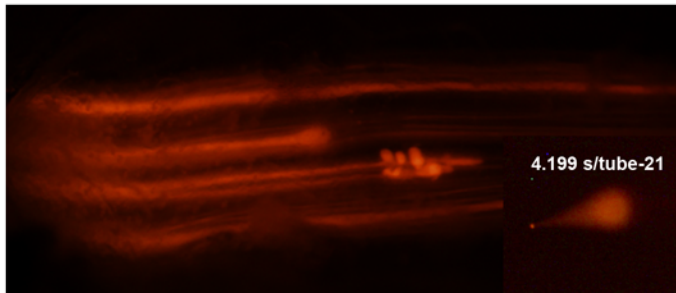

# Refracting tips (5)

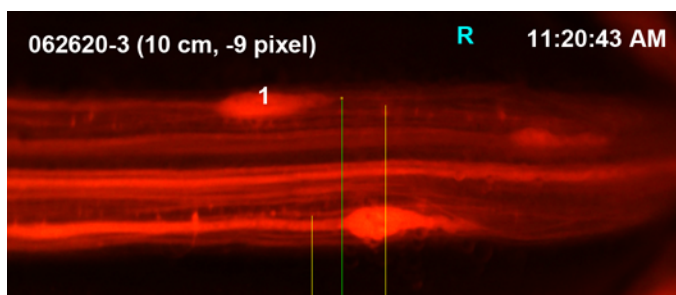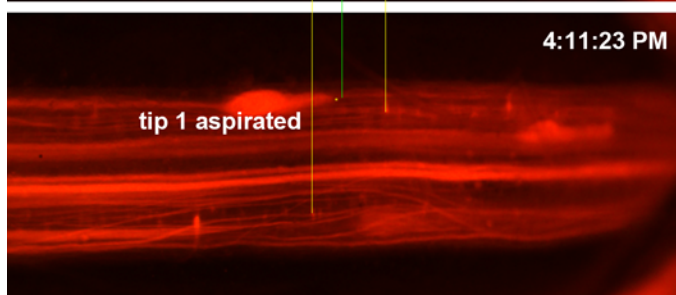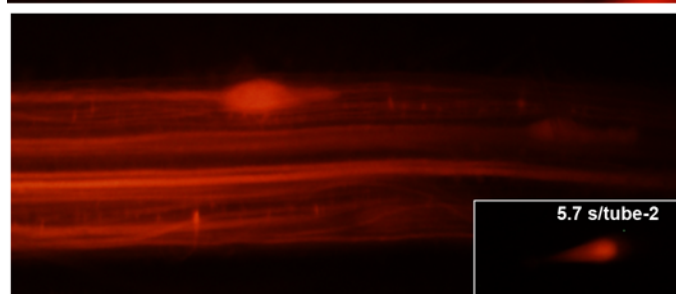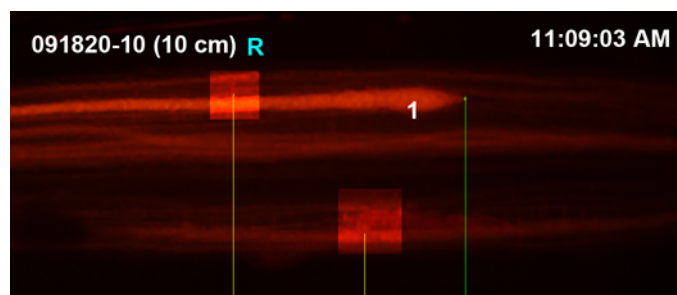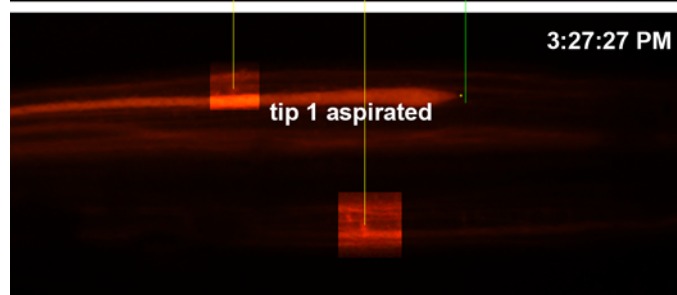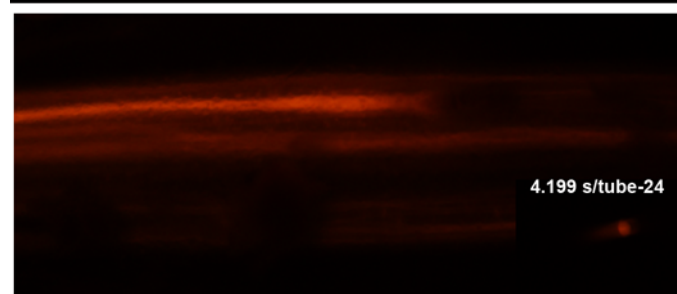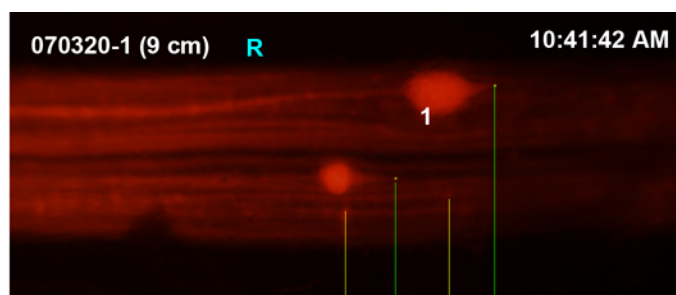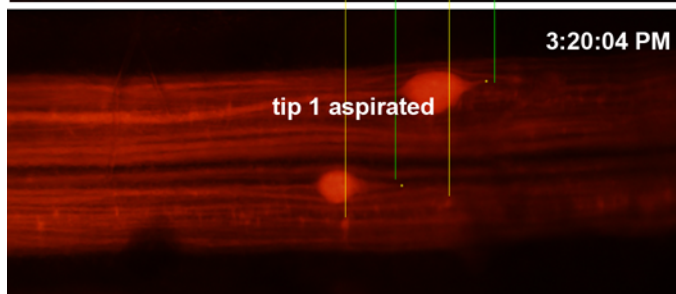

3.623 s/tube-7

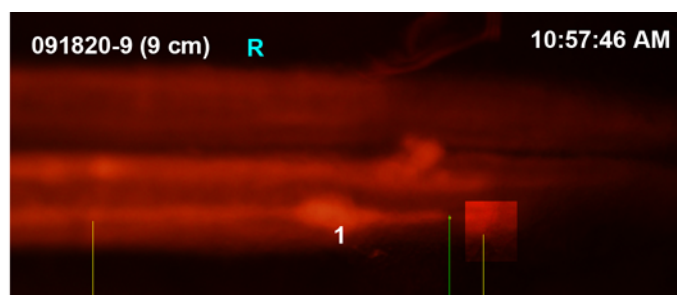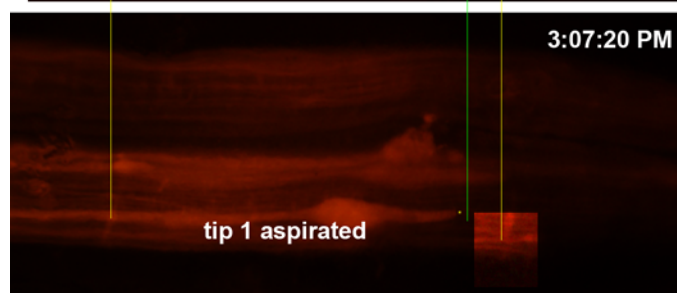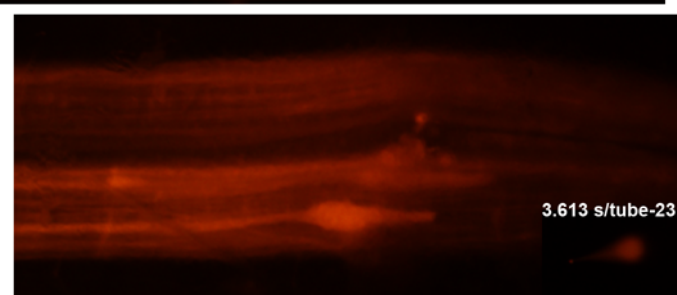

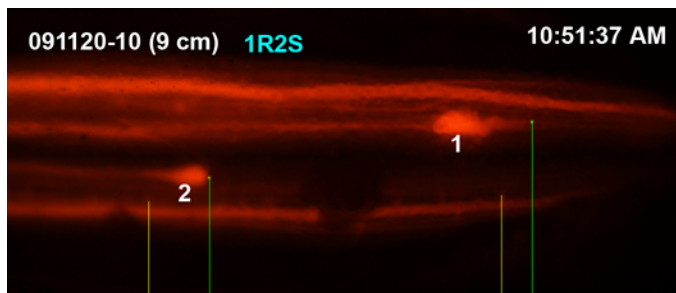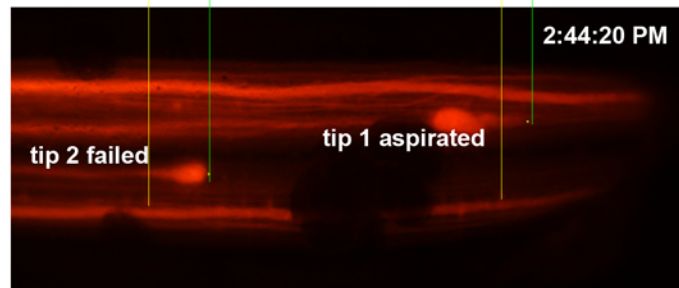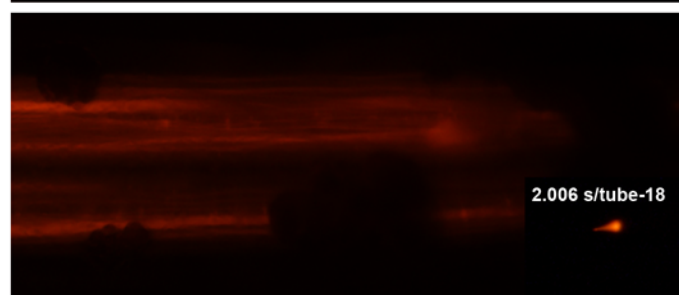

## Removed Samples (9)

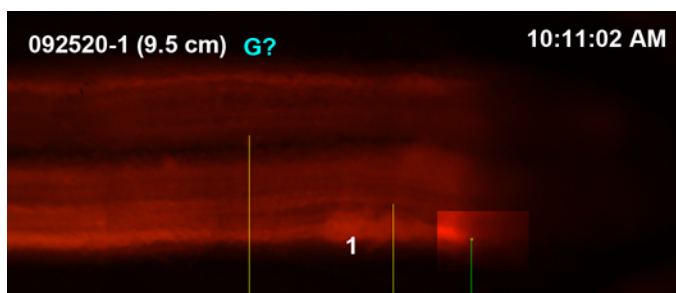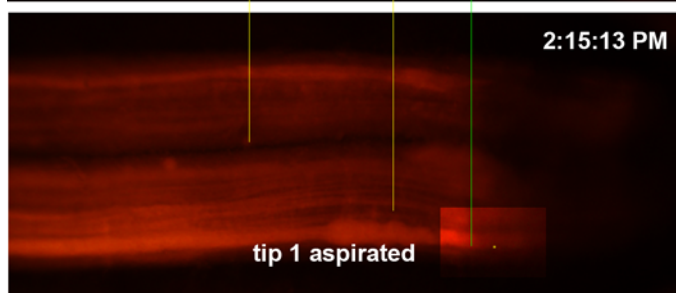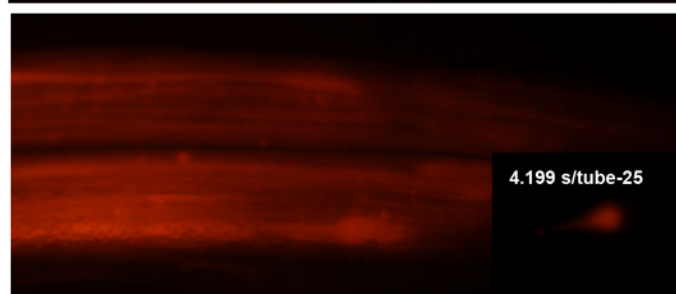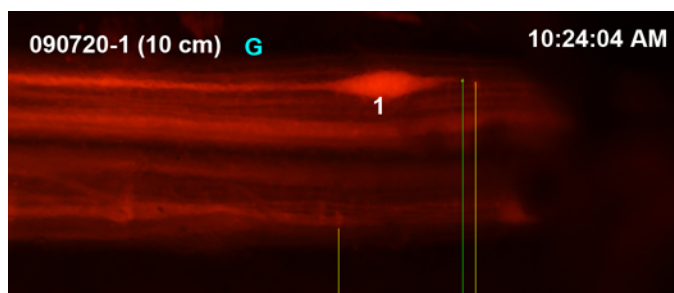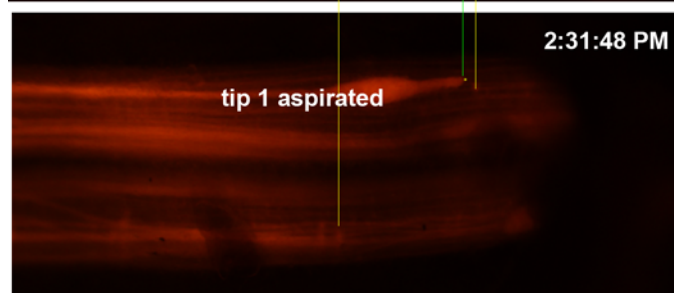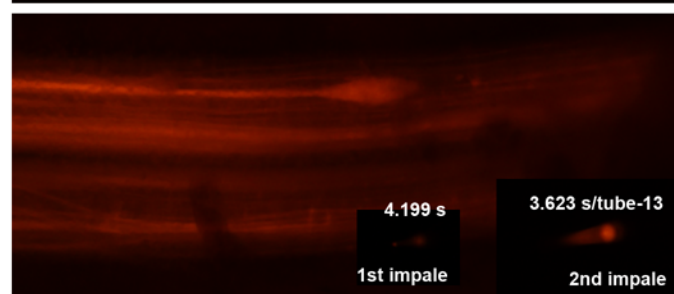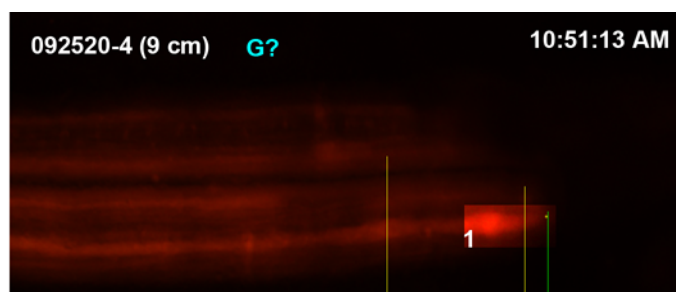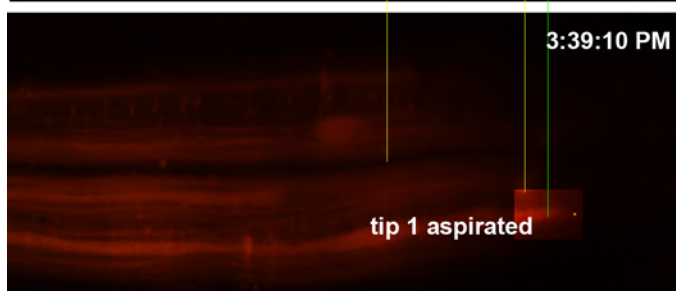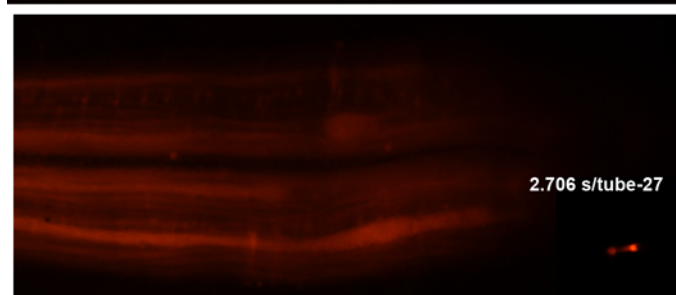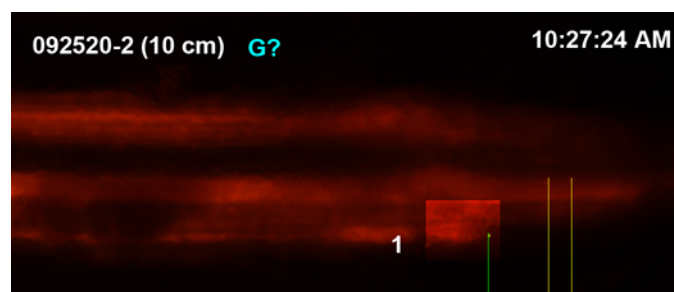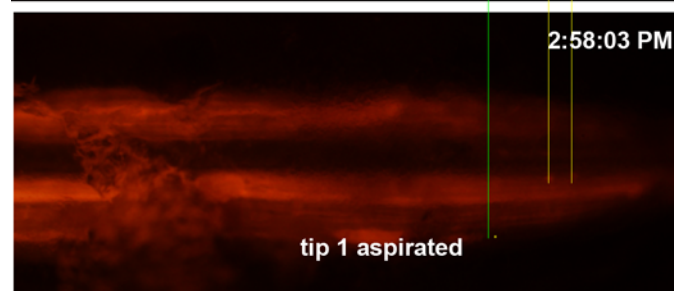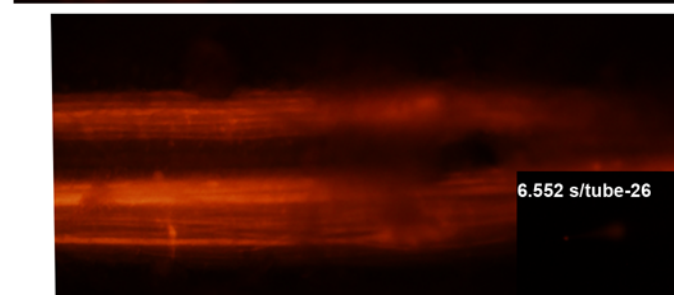

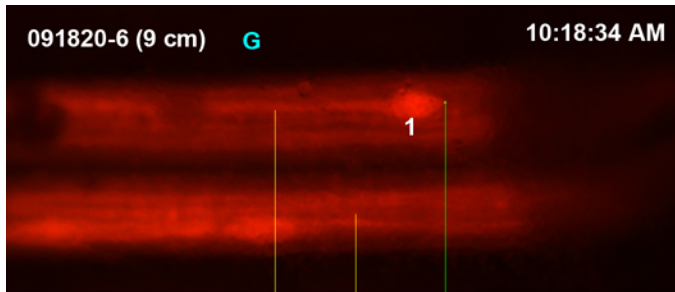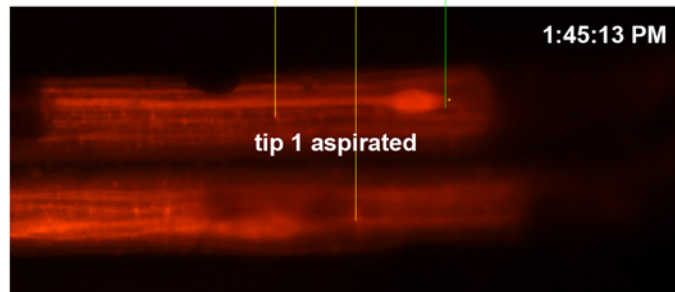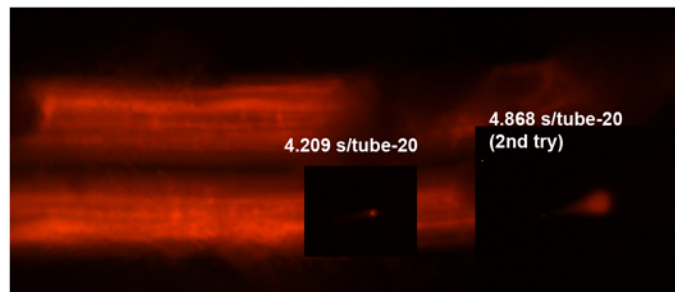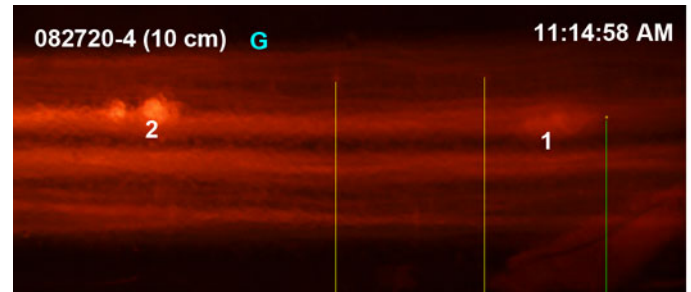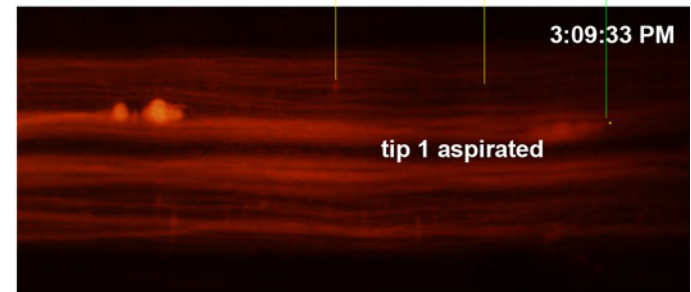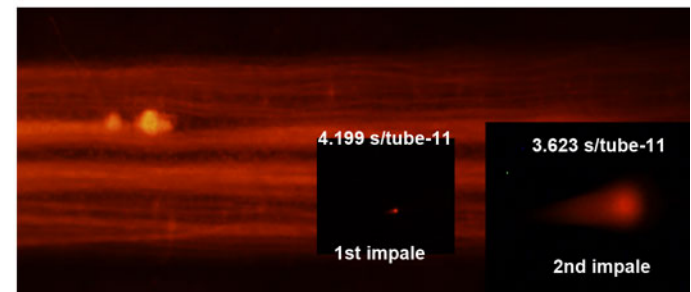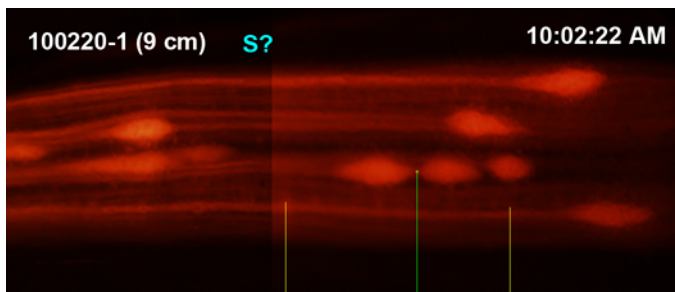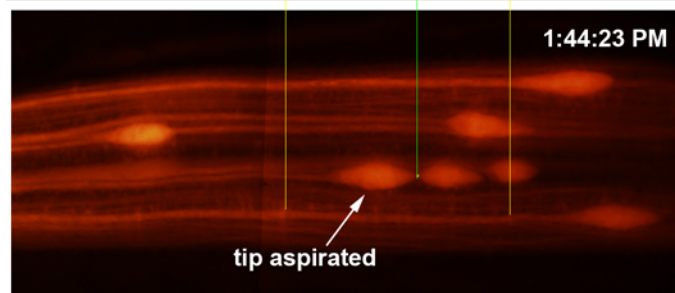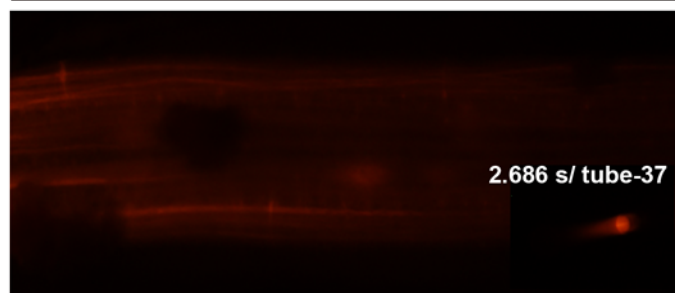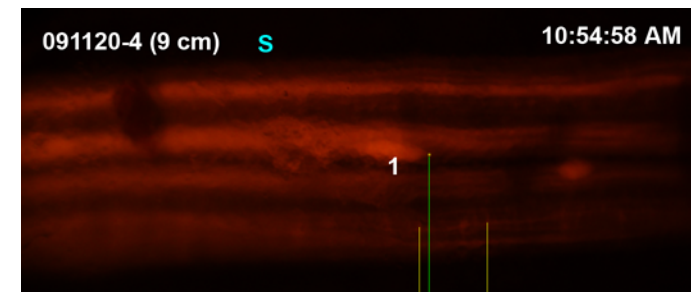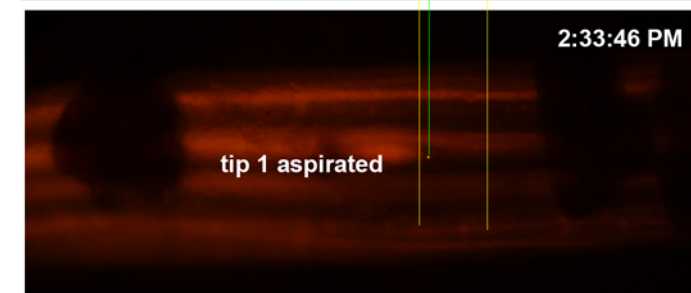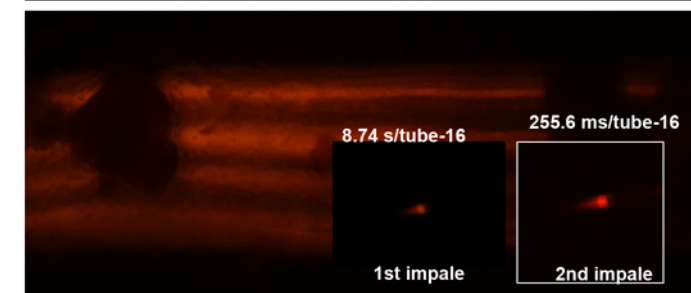

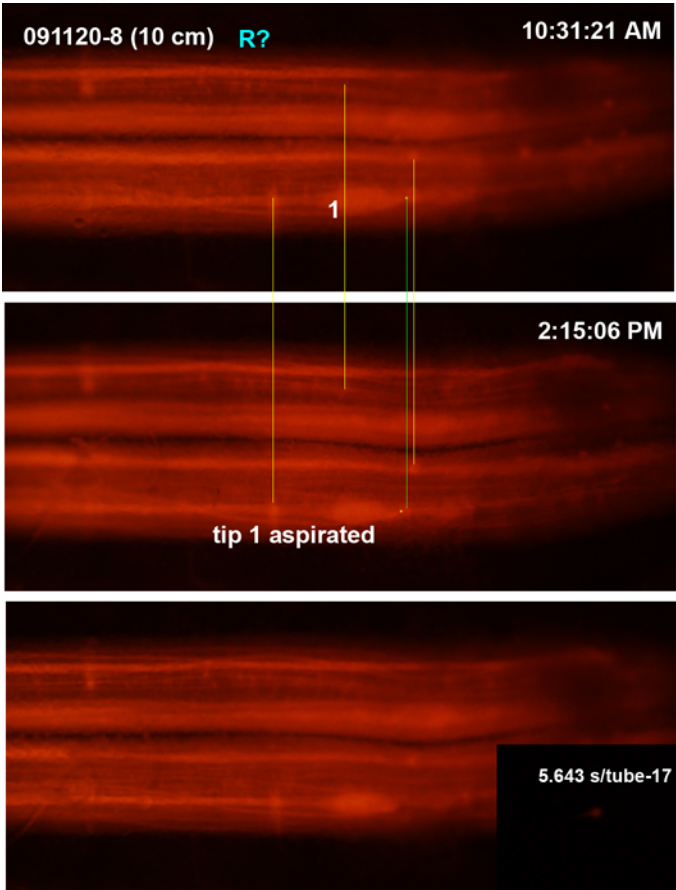

Supplement: Supplementary file 1 [file cells-11-02320-s001.zip › cells-1803217-supplementary/cells-1803217supplementary/cells-1803217-figures and tables/Figure S1 (spinal cord).pdf]

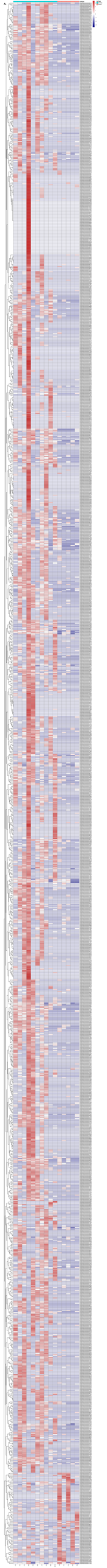

B.

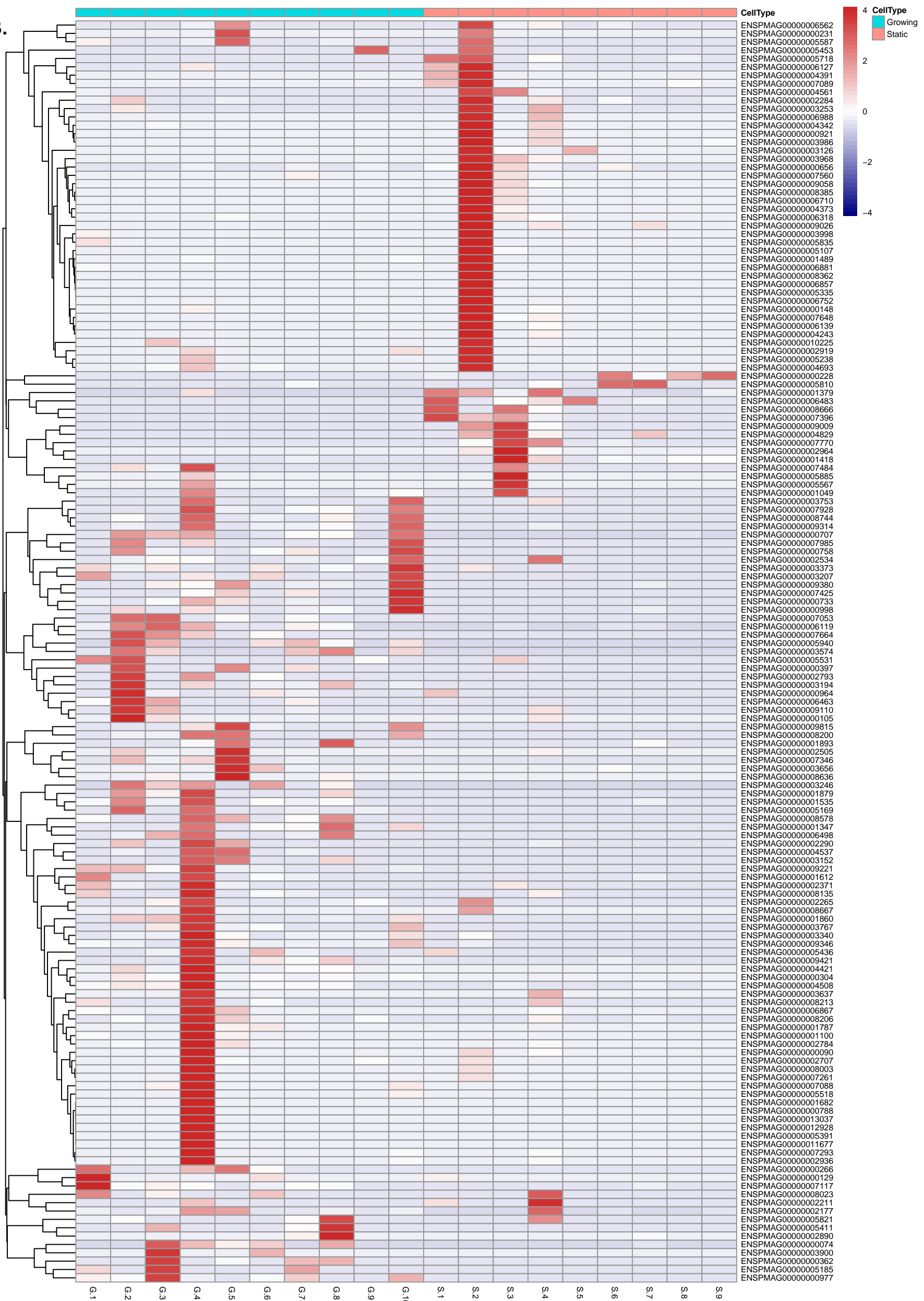

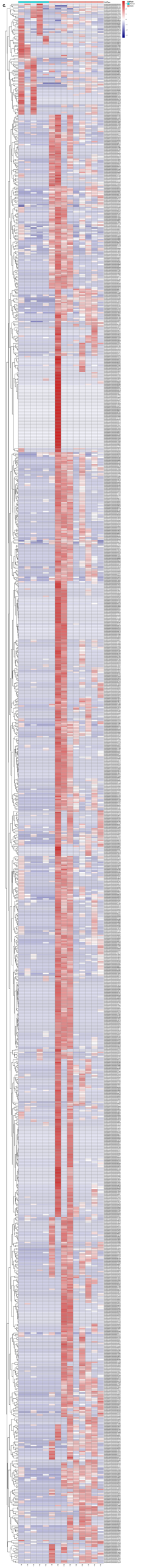

Supplement: Supplementary file 1 [file cells-11-02320-s001.zip › cells-1803217-supplementary/cells-1803217supplementary/cells-1803217-figures and tables/Figure S2-(heat maps).pdf]

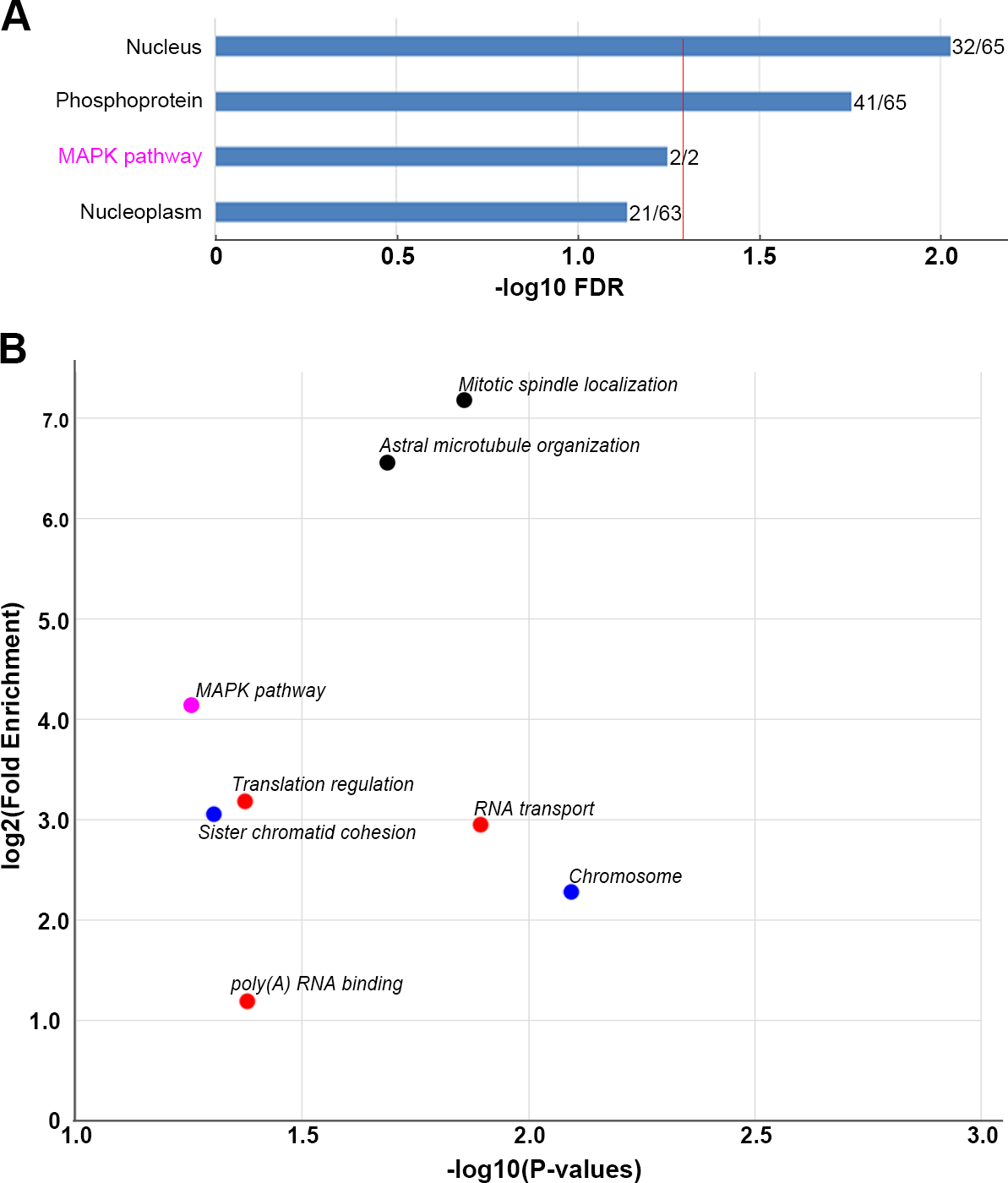

Supplement: Supplementary file 1 [file cells-11-02320-s001.zip › cells-1803217-supplementary/cells-1803217supplementary/cells-1803217-figures and tables/Figure S3 (G vs S).tif]

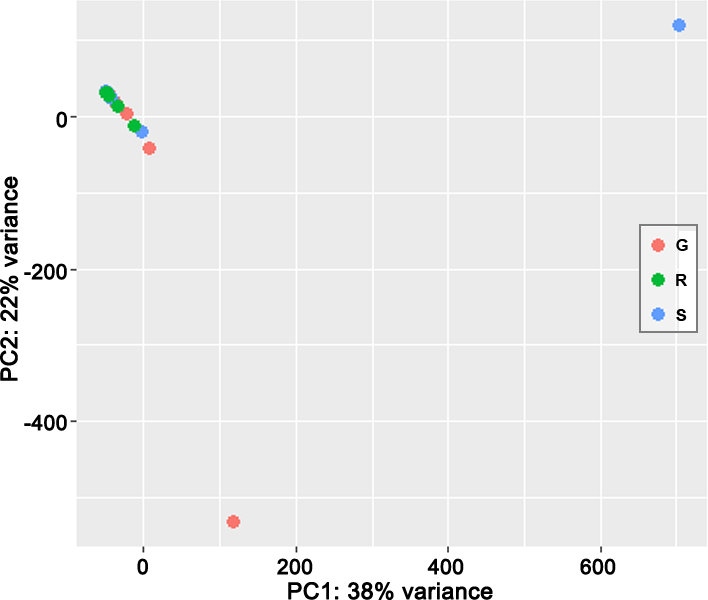

Supplement: Supplementary file 1 [file cells-11-02320-s001.zip › cells-1803217-supplementary/cells-1803217supplementary/cells-1803217-figures and tables/Figure S4 (PCA).tif]

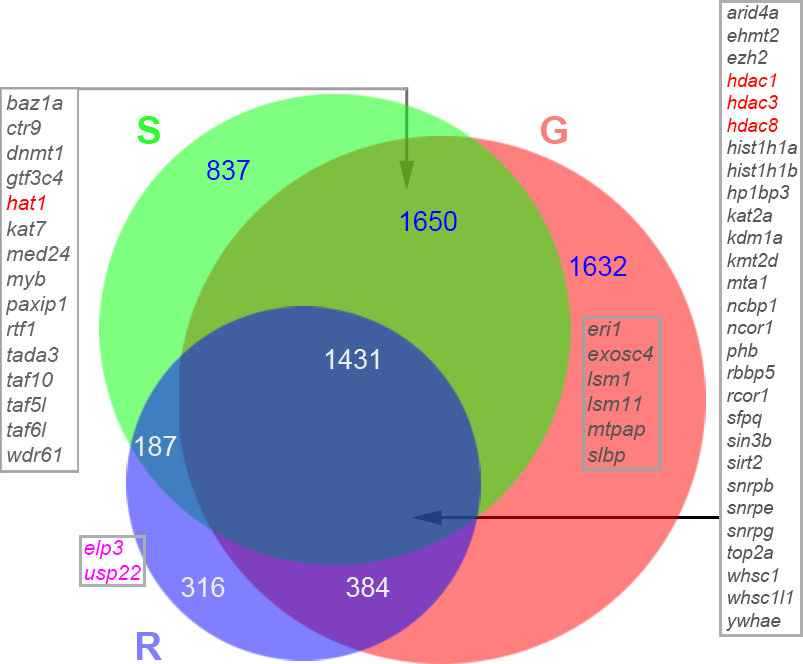

Supplement: Supplementary file 1 [file cells-11-02320-s001.zip › cells-1803217-supplementary/cells-1803217supplementary/cells-1803217-figures and tables/Figure S5 (Venn-diagram-histone).tif]

**map3k2**

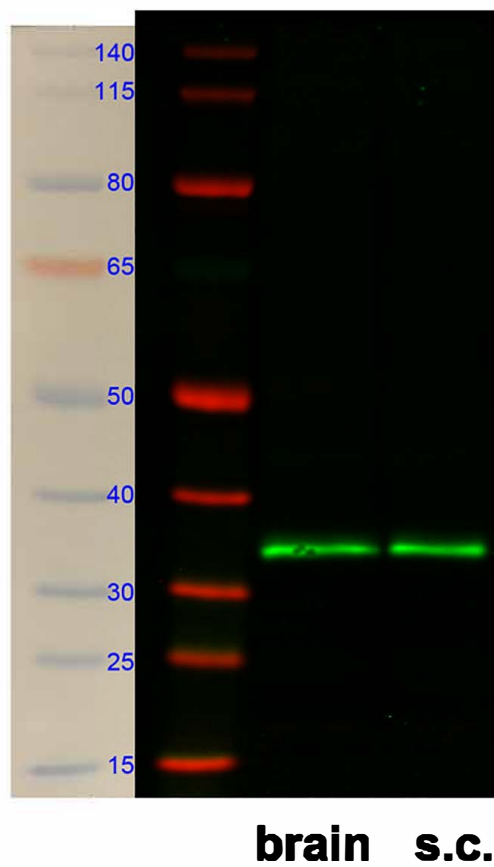

**csnk1e**

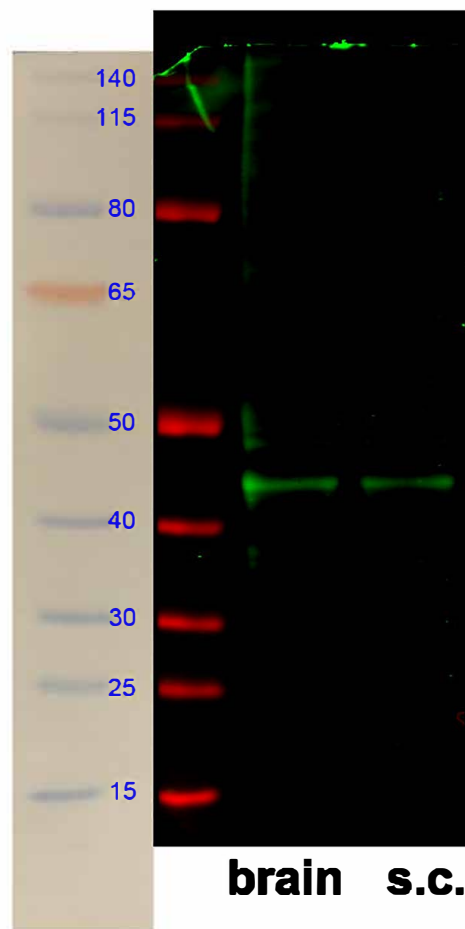

Supplement: Supplementary file 1 [file cells-11-02320-s001.zip › cells-1803217-supplementary/cells-1803217supplementary/cells-1803217-figures and tables/Figure S6 (Western blot).pdf]

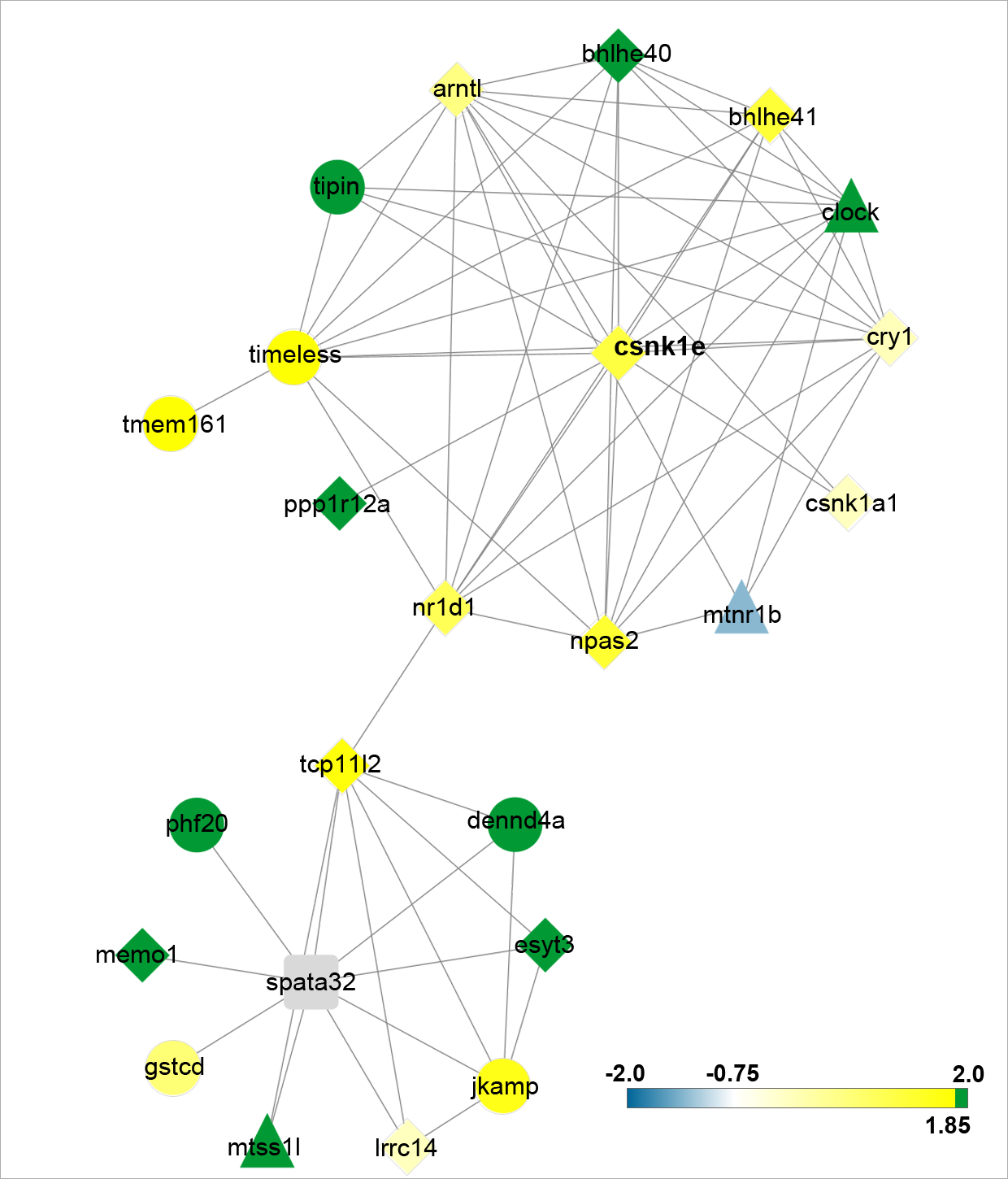

Supplement: Supplementary file 1 [file cells-11-02320-s001.zip › cells-1803217-supplementary/cells-1803217supplementary/cells-1803217-figures and tables/Figure S7 (PPI-csnk1e).tif]
